# Predicting Binding Free Energies of PDE2 Inhibitors. The Difficulties of Protein Conformation

**DOI:** 10.1038/s41598-018-23039-5

**Published:** 2018-03-20

**Authors:** Laura Pérez-Benito, Henrik Keränen, Herman van Vlijmen, Gary Tresadern

**Affiliations:** 1 Computational Chemistry, Janssen Research & Development, Janssen Pharmaceutica N. V., Turnhoutseweg 30, B-2340 Beerse, Belgium; 2 Present Address: Computational Chemistry and Struct. Biol., H. Lundbeck A/S, Ottiliavej 9, 2500 Valby, Denmark

## Abstract

A congeneric series of 21 phosphodiesterase 2 (PDE2) inhibitors are reported. Crystal structures show how the molecules can occupy a ‘top-pocket’ of the active site. Molecules with small substituents do not enter the pocket, a critical leucine (Leu770) is closed and water molecules are present. Large substituents enter the pocket, opening the Leu770 conformation and displacing the waters. We also report an X-ray structure revealing a new conformation of the PDE2 active site domain. The relative binding affinities of these compounds were studied with free energy perturbation (FEP) methods and it represents an attractive real-world test case. In general, the calculations could predict the energy of small-to-small, or large-to-large molecule perturbations. However, accurately capturing the transition from small-to-large proved challenging. Only when using alternative protein conformations did results improve. The new X-ray structure, along with a modelled dimer, conferred stability to the catalytic domain during the FEP molecular dynamics (MD) simulations, increasing the convergence and thereby improving the prediction of ΔΔG of binding for some small-to-large transitions. In summary, we found the most significant improvement in results when using different protein structures, and this data set is useful for future free energy validation studies.

## Introduction

The accurate prediction of protein ligand binding affinities is of high interest for drug discovery^1^. Free-energy simulations provide a rigorous approach and methods such as free-energy perturbation (FEP) make use of computational molecular dynamics (MD) simulations to compute the free-energy difference between two structurally related ligands^2^. The theory and application dates back several decades^3–9^. There is a resurgence of interest due to improved force fields, new sampling algorithms, and low-cost parallel computing often using graphics processing units (GPU)^10–12^ and modern implementations of these approaches have emerged^13,14^. The turnaround time is now sufficiently short that calculated binding affinities can impact drug discovery^15^.

Drug discovery lead optimisation (LO) requires synthesising analogues of important compounds. Hence, computation of accurate relative binding affinities is well suited. Given the technological advancements and high interest it is no surprise that applications are emerging^16–24^. Recent work from our labs^25–27^ showed good performance of FEP at predicting the binding energy of BACE-1 inhibitors, with mean unsigned error (MUE) between calculation and experiment <1 kcal/mol. However, outliers arise due to insufficient sampling: either in regions where ligands interact with flexible loops of the protein, or due to inconsistent movements between repeats or similar perturbations. Where significant ligand-induced protein reorganisation is required sampling needs to be increased (up to 50 ns per λ window) and replica exchange with solute tempering (REST) should be extended to include protein residues^28^. Inspired by the recent Lim *et al*. report and our interest to understand the ‘domain of applicability’ of FEP, here we study phosphodiesterase 2 (PDE2) inhibitors that require protein flexibility to bind.

**Figure 1:**
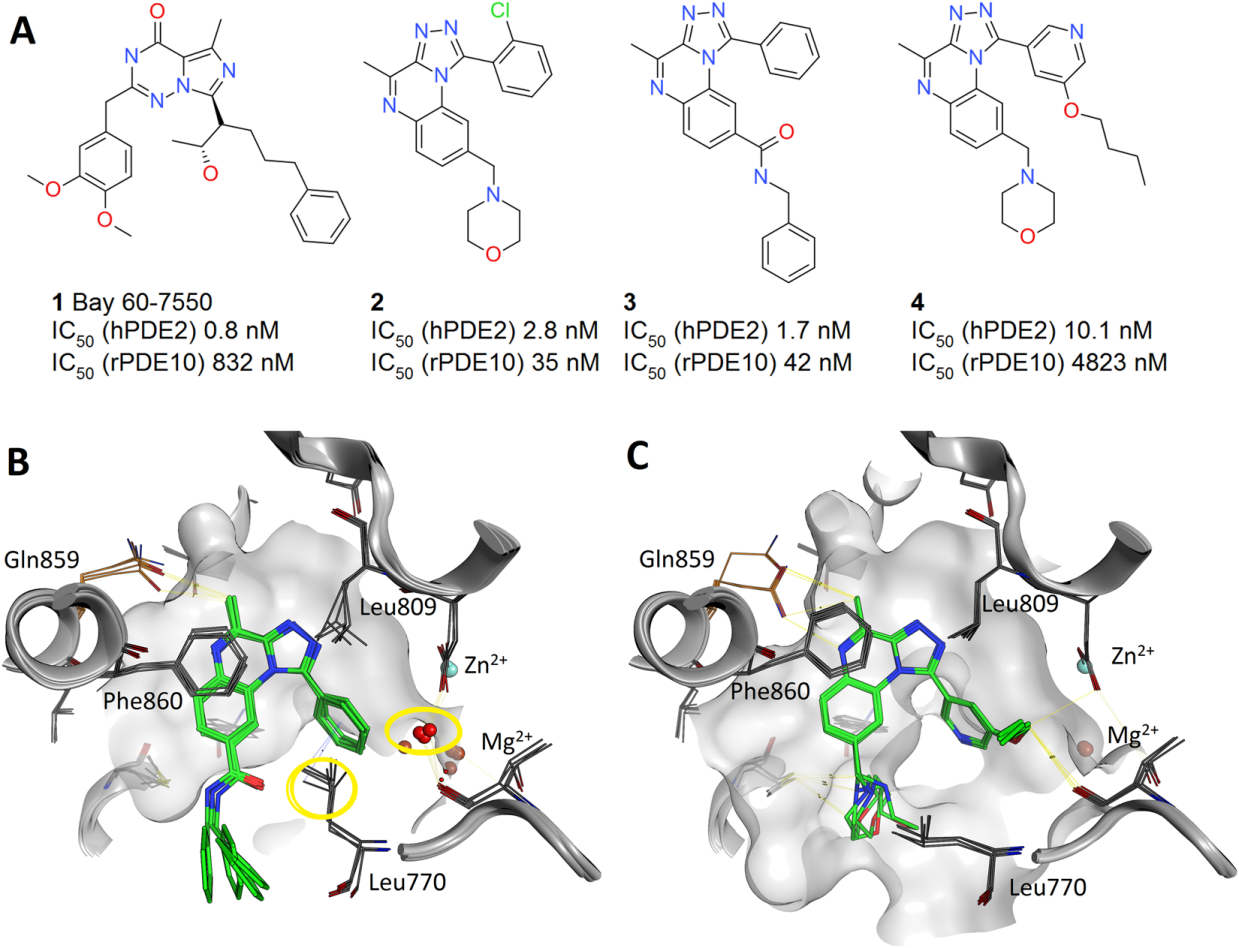
(**A**) PDE2 inhibitors **1** to **4**. (**B**) Crystal structure binding mode for molecule **3** (PDB 4D09) and (**C**) crystal structure of **4** (PDB 4D08). The superposition of the 4 chains in the crystallographic asymmetric unit is shown for each ligand. The waters that are displaced and the Leu770 that moves outwards are highlighted in yellow circles, the highlighted water position in panel B corresponds to the top-pocket.

PDE2 is a dual-substrate enzyme, degrading both cAMP and cGMP, and is predominantly expressed in the brain. It is the most abundant PDE subtype in the hippocampus, a brain structure playing an important role in cognitive process and cyclic nucleotide signaling which is implicated in neuronal plasticity and memory. Therefore, PDE2 inhibition is indicated in central nervous system disorders such as Alzheimer’s disease^29^. The first reported high affinity and selective PDE2 inhibitor was Bay60–7550, **1** Fig. 1. The selectivity of **1** arises from the unusual phenpropyl group entering a ‘top-pocket’ above the active site^30,31^. We synthesized a series of tricyclic inhibitors to target the same top-pocket. Selectivity was improved and the binding mode of **3** and **4** was confirmed by X-ray crystallography. Molecule **3** (PDB 4D09) does not enter the top-pocket and Leu770 maintains a closed conformation (χ_1_ angle −68°) and the top-pocket contained two water molecules. However, **4** (PDB 4D08), with an O-Bu substituent, enters the pocket displacing the two water molecules and Leu770 adopts a new outward orientation (χ_1_ 180°), Fig. 1^30^. Hence, analogous to simpler test cases^32^, we observe a change in rotameric state from gauche to trans of an amino acid side chain in the presence of different ligands in tandem with water displacement. Here we aim to retrospectively predict this top-pocket binding event using FEP calculations.

As well as the small-scale binding site motions that open and close the top-pocket, we also focus on another aspect of protein dynamics, namely large-scale conformational change. Via crystallography we disclose a previously unreported conformational state of the PDE2 catalytic domain. We call this an intermediate H-loop conformation, and show how this large-scale change of H-loop from open to intermediate states affects the calculation of binding free energies. Therefore, this is an attractive study: i) test conformational changes at different scales supported with X-ray structures, ii) perturbations can be relatively small amongst the 21 close analogues with incremental increases in substituent size, iii) a greater than 3 kcal/mol range of activity, iv) water structure adaptation. In summary, we apply FEP for predicting the binding free energies of this LO test case. Multiple protocols are explored, including improving sampling, averaging results, attention to local water structure, etc. The work reveals that the most obvious protein conformation may not be the best for free energy calculations. Meanwhile, the overall difficulties to predict affinity for this real-life scenario demonstrate that effort is needed to improve and diagnose FE calculations.

## Results

### Conformational states of PDE2 catalytic domain

**Figure 2:**
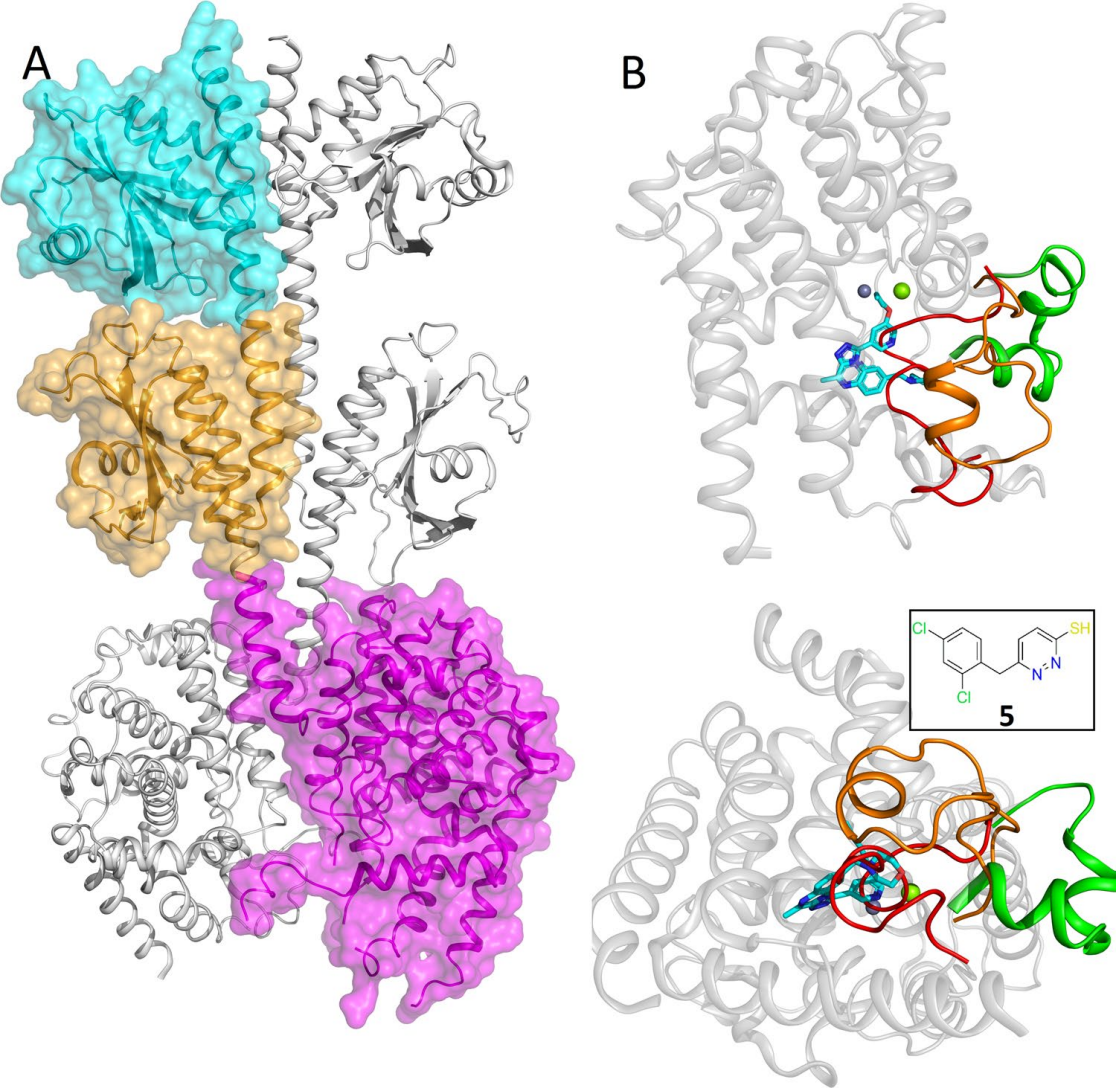
Conformational states of PDE2 catalytic domain. (**A**) The near full length inactive PDE2 dimer, one monomer shown with surface and the other without, the catalytic domains are adjacent and one is shown with a magenta surface. (**B**) Superposition of catalytic domains showing distinct H-loop conformations. The inactive H-loop structure in red, H-loop fully open in green, our new X-ray structure with molecule **5** (inset) showing intermediate H-loop in orange.

During our search for PDE2 inhibitors we applied a fragment approach and obtained a crystal structure of PDE2 with fragment **5**, Fig. 2. The crystals contained one monomer of PDE2A in the asymmetric unit. The structure had a good resolution of 1.5 Å and the electron density shows an unambiguous binding mode, Figure S1. The pyridazine hydrogen bonded to key amino acid Gln859 and formed a pi stacking interaction with Phe862. The new crystal structure showed a previously unseen conformational state of the protein. The H-loop (702–728) covers the ligand in the binding site in contrast to entirely open H-loop conformations typically observed in published PDE2A catalytic domain crystal structures (Fig. 2 and S2). The near full length (FL, residues 215–900) PDE2 crystal structure^33^ revealed an inactive dimeric state with the H-loop filling the active site of each monomer preventing substrate or small molecule binding. Hence, the new crystal structure provides a snapshot of an intermediate H-loop conformation between the inactive (active site blocked) and fully open states (as seen in 4D08 and 4D09).

### PDE2 inhibitors and bioactivity

The synthesis of the 21 PDE2 inhibitors, Table 1, followed previously reported synthetic routes^25,30^. The molecules contain small differences in the **R**-group on the (hetero)aromatic ring in the 1 position of the 4-methyl-[1,2,4]triazolo[4,3-*a*]quinoxaline scaffold. Also, different halogen atoms on the same ring, or modification of the ring from phenyl to pyridyl are included. The tricyclic scaffold binds in the hydrophobic clamp between Phe862 and Phe830 and H-bonds with Gln859. The morpholino substituent is solvent exposed. The R-group in this series explores the top-pocket formed by hydrophobic residues Leu770, Leu809, Ile866, and Ile870. Leu770 shows conformational change if the pocket is occupied by parts of a ligand, as seen in Fig. 1. The 4D09 structure contains two water molecules in the top-pocket that are displaced by the *n*-butyl chain in 4D08 structure. In general, the small and large molecules are both highly active, whereas there is an energetic cost for partially occupying the top-pocket, with intermediate inhibition activities seen.

**Table 1:** PDE2 inhibitors studied.


**Comp.#**	**pIC**_**50**_^a^	***n*** ^**b**^	**R**	**W**	**X**	**Y**	**Z**
**2**	8.57 ± 0.15	8	H	H	Cl	C	H
**4**	8.14 ± 0.30	13	OBu	H	H	N	H
**6**	8.02 ± 0.20	5	H	H	Cl	C	F
**7**	8.71 ± 0.19	13	H	F	Cl	C	H
**8**	7.59 ± 0.23	3	H	H	Me	N	H
**9**	8.22 ± 0.30	9	OMe	H	Cl	C	H
**10**	7.92 ± 0.08	3	OMe	H	H	N	H
**11**	8.21 ± 0.21	4	OEt	H	Cl	C	H
**12**	7.37 ± 0.20	3	OEtF	H	H	N	H
**13**	8.19 ± 0.17	4	OPr	H	Cl	C	H
**14**	7.52 ± 0.16	5	OPr	H	H	N	H
**15**	8.75 ± 0.36	6	O*i*Pr	H	Cl	C	H
**16**	6.42 ± 0.13	6	O*i*Pr	H	H	N	H
**17**	7.53 ± 0.15	3	MeOEt	H	H	N	H
**18**	7.05 ± 0.25	7	EtOMe	H	H	N	H
**19**	7.19 ± 0.01	2	OPrF	H	H	N	H
**20**	7.0 ± 0.19	5	OEtOMe	H	H	N	H
**21**	8.1 ± 0.22	8	OBu	H	Cl	C	H
**22**	6.93 ± 0.05	2	OBu	H	H	N	Cl
**23**	6.88 ± 0.31	2	PrOMe	H	H	N	H
**24**	7.85 ± 0.30	8	OMe-cycloPr	H	H	N	H

^a^Errors are standard deviations. ^b^*n* refers to number of independent repeat experimental measurements of pIC_50_, each repeat was performed in triplicate. The small compounds were: **2**, **6**, **7**, **8**, **9**, and **10**, and the large compounds were: **4**, **11**, **12**, **13**, **14**, **15**, **16**, **17**, **18**, **19**, **20**, **21**, **22**, **23** and **24**.

### Free energy calculations, FEP

#### H-loop open protein structures

To predict the activity of the compounds in Table 1 we began using the PDE2 crystal structures 4D09 and 4D08 solved with molecules **3** and **4**. All calculations used the same network of 34 perturbations (Figure S3) and began with 1 ns simulations per λ window, and 12 λ windows per perturbation in solvent and complex. In short, no immediate correlation was seen between calculation and experiment, Table 2. Increasing simulation time to 5 and 40 ns per λ window made no impact on ΔG (as evaluated by MUE with experiment). Repeats with new random seeds and averaging results also had no effect. With errors of 1.2–1.4 kcal/mol the calculations would not be useful for molecular design. Standard docking and MM/GBSA approaches showed worse performance. Docking with 4D09 failed for multiple large molecules and for 4D08 was anticorrelated with experimental activity. Meanwhile the best MM/GBSA approach had an MUE of 6.94 ± 3.74 kcal/mol and R^2^ of 0.08, Table S3 and Figure S4.

**Table 2:** Comparison of FEP and experimental predicted ΔG’s and ΔΔG’s (kcal/mol) for different attempted protocols and input protein structures.

Starting structure^a^	λ time (ns)^b^	n^c^	Extra features	ΔG All 21 molecules			MUE ΔG small molecules	MUE ΔG large molecules	MUE ΔΔG			
				MUE^d^	R^2^	SD^e^			All	Small-small	Large-large	Small-large
4D09	1	1	—	1.46 (±0.53)	0.13	—	2.15 (±1.02)	1.18 (±0.61)	1.56 (±0.59)	0.96 (±0.90)	1.26 (±0.52)	3.63 (±1.70)
4D08	1	1	—	1.20 (±0.47)	0.03	—	1.97 (±0.78)	0.89 (±0.44)	1.13 (±0.45)	0.57 (±0.65)	0.86 (±0.28)	3.04 (±1.22)
4D09	1	3	—	1.45 (±0.57)	0.08	0.17	2.11 (±0.91)	1.18 (±0.64)	1.50 (±0.61)	1.07 (±0.71)	1.04 (±0.52)	3.76 (±1.79)
4D08	1	3	—	1.33 (±0.49)	0.04	0.44	2.01 (±0.68)	1.06 (±0.55)	1.22 (±0.51)	0.58 (±0.40)	0.85 (±0.33)	3.45 (±1.39)
4D09	5	1	—	1.36 (±0.57)	0.13	—	2.13 (±1.02)	1.14 (±0.66)	1.50 (±0.61)	1.15 (±0.95)	1.17 (±0.52)	3.72 (±1.91)
4D08	5	1	—	1.34 (±0.54)	0.01	—	2.16 (±0.63)	1.02 (±0.59)	1.20 (±0.51)	0.53 (±0.34)	0.92 (±0.26)	3.40 (±1.71)
4D09	5	3	—	1.41 (±0.58)	0.08	0.11	2.14 (±0.99)	1.11 (±0.63)	1.50 (±0.59)	1.10 (±0.90)	1.07 (±0.52)	3.64 (±1.70)
4D08	5	3	—	1.34 (±0.59)	0.00	0.18	2.28 (±0.73)	0.96 (±0.61)	1.20 (±0.52)	0.59 (±0.37)	0.81 (±0.26)	3.53 (±1.54)
4D09	40	1	—	1.44 (±0.62)	0.06	—	2.21 (±1.03)	1.13 (±0.69)	1.53 (±0.60)	1.20 (±0.85)	1.15 (±0.52)	3.69 (±1.93)
4D08	40	1	—	1.23 (±0.54)	0.03	—	1.91 (±0.60)	0.95 (±0.64)	1.22 (±0.51)	0.65 (±0.42)	0.96 (±0.37)	3.15 (±1.86)
4D09	5	1	Leu770 REST	1.44 (±0.58)	0.07	—	2.12 (±0.96)	1.17 (±0.65)	1.59 (±0.62)	1.12 (±0.89)	1.23 (±0.56)	3.81 (±1.66)
4D08	5	1	Leu770 REST	1.30 (±0.52)	0.02	—	2.04 (±0.59)	0.99 (±0.59)	1.17 (±0.48)	0.53 (±0.26)	0.89 (±0.28)	3.24 (±1.54)
4D08	5	1	Leu770 H_2_O^f^	1.18 (±0.52)	0.05		1.81 (±0.80)	0.93 (±0.59)	1.30 (±0.56)	0.81 (±0.68)	0.99 (±0.43)	3.29 (±1.86)
4D09	5	1	GCMC H_2_O	1.43 (±0.64)	0.06	—	2.21 (±0.94)	1.12 (±0.73)	1.51 (±0.62)	1.05 (±0.94)	1.14 (±0.48)	3.72 (±2.27)
4D08	5	1	GCMC H_2_O	1.16 (±0.50)	0.02	—	1.95 (±0.60)	0.85 (±0.53)	1.06 (±0.48)	0.52 (±0.35)	0.76 (±0.31)	3.05 (±1.53)

^a^Initial protein structure used for FEP calculations. ^b^Simulation time for each λ window. ^c^Number of repeats. ^d^Mean unsigned error in kcal/mol compared to experiment after mean-centred fitting, numbers in parentheses are 99% confidence interval. ^e^Standard deviation in MUE if repeat calculations were performed. ^f^Leu770 H_2_O protocol refers to leaving the Leu770 top-pocket waters in place despite clashes with large ligands.

**Figure 3:**
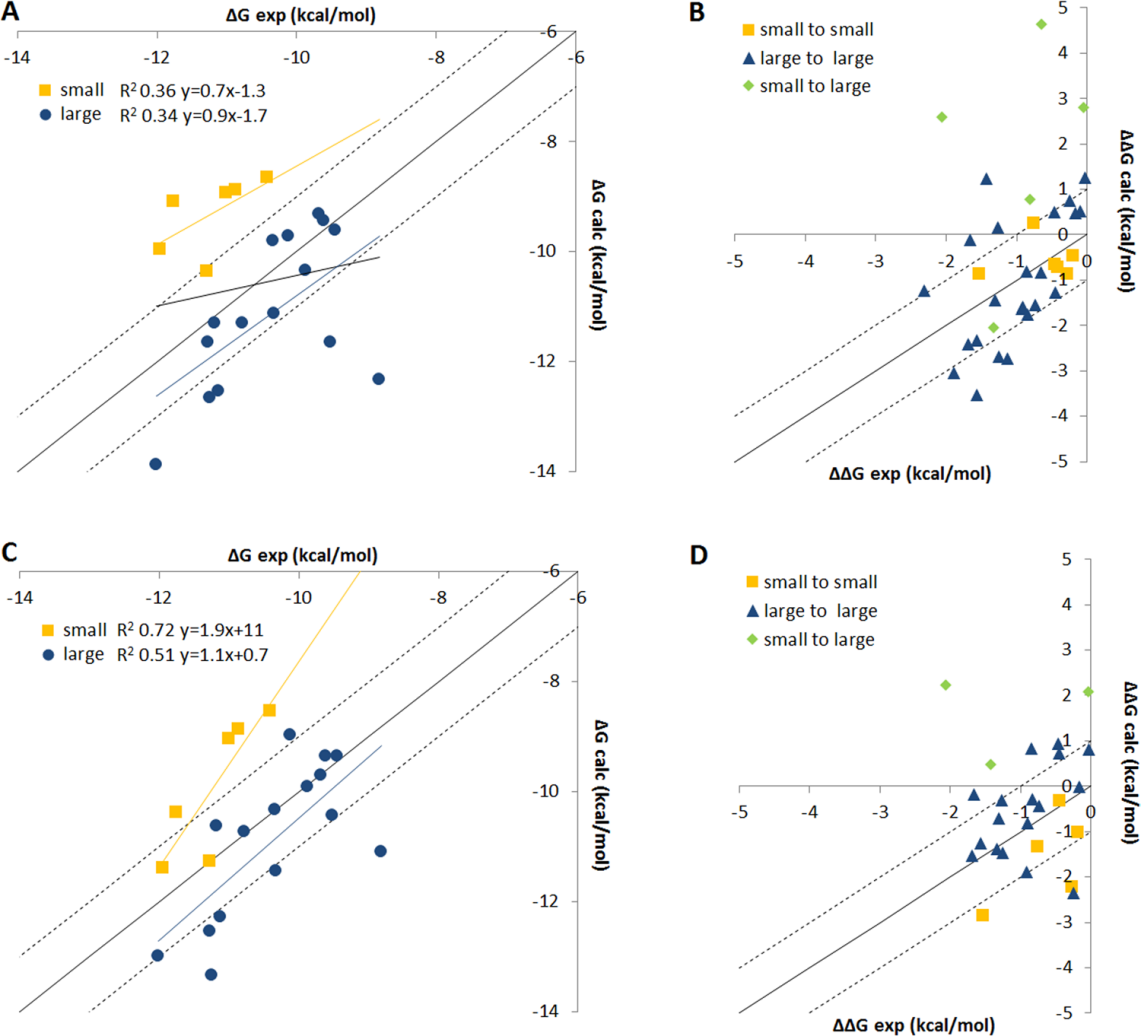
Comparing ΔG and ΔΔG for FEP protocols performed with 4D08 starting structure (**A** and **B**) compared to dimer model structure (**C** and **D**). Both protocols run with 40 ns λ window simulation time.

For the FEP, large compounds were better predicted than smaller examples. With a single 5 ns FEP simulation using the 4D08 structure the MUE for large compounds was 1.02 ± 0.59 versus 2.16 ± 0.63 kcal/mol for small. Figure 3 shows the correlation plot of ΔG and ΔΔG with experiment, illustrating the inaccurate prediction of ΔG for the small molecules. The MUE of the ΔΔG for each perturbation were in a similar range, 1.20 ± 0.51 kcal/mol for all perturbations in the single 4D08 5 ns protocol. However, transitions between molecules of similar size were treated much better, ΔΔG MUE for small-to-small and large-to-large transitions were 0.53 ± 0.34 and 0.92 ± 0.26 kcal/mol respectively, whereas for perturbations of small-to-large the MUE was always >3 kcal/mol. The high MUE in ΔG for small molecules originated from the inaccurate ΔΔG of small-to-large perturbations. No improvements could be made by either: 1) doubling the number of perturbations; 2) removing perturbations with more than three heavy atom changes; 3) using the LOMAP tool^34^ to generate a more conservative set of 25 perturbations; 4) doubling the number of λ windows to 24 per perturbation (ΔΔG for small-to-large changes MUE 3.34 ± 1.82 kcal/mol), see supplementary information Figures S5 to S7. Only removing all the small or large molecules improved results. For the 4D08 starting structure the MUE for ΔG was 0.65 ± 0.51 and 1.05 ± 0.61 kcal/mol for small and large compounds respectively. Unfortunately, this solution is unsatisfactory for prospective modelling because it would not be possible to rank small molecules alongside larger ones.

Surprisingly given the Leu770 movement, sampling made no impact on results. Analysing the trajectories revealed Leu770 mostly sampled the open state for the smaller compound **2**, whilst for the larger compound **21** both rotamers were sampled, Figure S8. Repeating the 5 ns protocol FEP calculations with Leu770 added into the REST region, showed no change in results. The overall ΔG MUE was 1.44 ± 0.58 and 1.30 ± 0.54 kcal/mol for 4D09 and 4D08 starting structures respectively, and the ΔG of the small molecules was still poorly predicted.

The crystal structure 4D09 with **3** contained two waters occupying the top-pocket and Leu770 in the closed conformation. A large ligand such as **4** displaces the waters. It seemed the waters could be key to accurately capture the free energy change from a small-to-large substituent. Firstly, considering differences in the binding site water network (not just in the top-pocket) we swapped the water conformations from one input structure to the other. For a single 5 ns per λ window FEP protocol there was no change in results, the MUE for the ΔG was 1.25 ± 0.50 kcal/mol for 4D09 protein structure with the 4D08 waters, and 1.25 ± 0.51 kcal/mol for the 4D08 protein structure with 4D09 waters (details not shown in Table 2). This is unsurprising because the water structures were similar and the top-pocket waters had been removed in both cases to avoid clashes. We considered if the waters were left in the top-pocket, could better results be attained despite the clashes with the large ligands? A 5 ns per λ window FEP protocol was performed but again with little impact on results, MUE for the ΔG and ΔΔG was 1.18 ± 0.52 and 1.30 ± 0.56 kcal/mol respectively and no improvement for the small-to-large ΔΔG transitions, with the MUE still >3 kcal/mol. We also used a Grand Canonical Monte Carlo (GCMC) simulation to place water molecules during the pre-production phase of the FEP MD simulations. The approach placed water in the top-pocket at the start of the simulations for the small molecules, but despite this it did not impact the results. The MUE for ΔG with the 4D09 starting structure was 1.43 ± 0.64 kcal/mol for 4D08 was 1.16 ± 0.5 kcal/mol. The apparent free movement of Leu770 during the simulations made the top-pocket relatively solvent accessible.

#### Alternative protein structures

As described, obvious sources of error such as local sampling, mapper setup, and water structure did not affect results. When examining the trajectories and the motion of Leu770 we noticed that its corresponding helix 12 (H12) moved outwards permitting the open and closed Leu conformations, Figure S9. The relatively small outward movement (~3 Å) occurred in the first few ns of all simulations. The helix then stabilized in an outward position not seen in crystal structures but due to its proximity we considered this might affect the perturbations. It was not possible to detect such a small movement in the overall protein RMSD and RMSF (root mean square fluctuation) plots (Figures S10 and S11 for 5 and 40 ns λ window protocols respectively using the 4D08 starting structure). Instead the plots were dominated by the large C-terminus movement, which started in a highly solvent exposed position (Figure S2), and moved substantially in the initial phases of simulations. The high protein RMSD (5–6 Å) was consistently seen regardless of whether the perturbation was between small-to-small, large-to-large or small-to-large molecules, hence the C-terminus instability was unlikely to be an important factor.

To further study these aspects of protein stability, firstly we performed additional equilibration. The penultimate step of the standard equilibration was 24 ps using NPT ensemble and constraints on solute (50 kcal/mol.Å^2^) and the last step released the restraints and ran for a further 240 ps. In this case we increased both of these steps to 5 ns, followed by the 5 ns production FEP MD. There was no change, H12 still moved outwards and using the 4D08 starting structure the ΔG and ΔΔG MUE for all molecules were 1.22 ± 0.58 and 1.18 ± 0.56 kcal/mol respectively, whilst the ΔΔG MUE for small-to-large perturbations was still 3.46 ± 2.18 kcal/mol (results not shown in Table 2). Concerned that the instability could not be overcome with moderately longer equilibration, we next performed a 100 ns classical MD simulation with molecule **21** and crystal structure 4D08, and started FEP calculations from the endpoint. The helix and C-terminus moved and then stabilized, but the subsequent FEP results were again similar to the single 5 ns per λ window results, with a MUE 1.2 ± 0.53 kcal/mol (not shown in Table 2). Hence preventing further movement of the C-terminus and H12 but stabilising them in experimentally unseen conformations was not beneficial for the binding energy predictions.

The experimental assay was performed on full length (FL) dimeric PDE2 hence we studied the near FL structure^33^ and noticed the interaction between catalytic domains. In fact, the M-loop (830–856) of one monomer is less than 5 Å from Leu770 of the other monomer (consider Asp835 and Lys838 to Leu770 distances). Hence stabilising effects between monomers are present in FL PDE2 but not in the typical monomeric catalytic domain structures. Ligand binding energies cannot be modelled with the FL structure because the active site is filled with the closed H-loop. Nevertheless, our structure of PDE2A catalytic domain solved with **5** had the H-loop folded over the binding site but allowed ligand placement for binding energy calculations. In addition, the folded H-loop contacts the bottom of H12 and could stabilize the helix.

This new structure was used for FEP calculations with the same 21 ligands. The ΔG MUE improved to 0.84 ± 0.35 for all ligands with a single 5 ns per λ window protocol, Table 3. The MUE for all ΔΔG calculations was also better, 0.89 ± 0.40 kcal/mol. Small-to-large perturbations were still the most problematic for the ΔΔG calculations, although the MUE was lower than previous calculations, 2.4 ± 1.51 kcal/mol. Analysis of the simulations showed that the new structure prevented H12 opening in some cases, Figure S9. Encouraged, we tested the stability of the results versus sampling time, performing 1, 5, and 40 ns λ windows FEP protocols, with the former two being performed in triplicate. The results were similar, with ΔG MUE being 0.99 ± 0.43 and 0.99 ± 0.41 kcal/mol for 1 and 40 ns λ window calculations again showing that extended sampling did not improve results. As previously, we compared with standard methods, docking and MM/GBSA both performed worse. Docking with the new structure was again inversely correlated with experimental activity and the best MM/GBSA protocol gave very high errors, MUE 10.40 ± 4.45 kcal/mol and no correlation, R^2^ 0.04.

**Table 3:** Comparison of FEP and experimental predicted ΔG’s and ΔΔG’s (kcal/mol) for different attempted protocols and input protein structures.

Starting structure^a^	λ time (ns)^b^	n^c^	Extra features	ΔG All 21 molecules			MUE ΔG small molecules	MUE ΔG large molecules	MUE ΔΔG			
				MUE^d^	R^2^	SD^e^			All	Small-small	Large-large	Small-large
New^*f*^	1	1	—	0.94 (±0.43)	0.01	—	1.50 (±0.68)	0.71 (±0.47)	1.00 (±0.39)	0.76 (±0.71)	0.74 (±0.35)	2.47 (±0.84)
New	1	3	—	0.85 (±0.44)	0.01	0.37	1.58 (±0.75)	0.56 (±0.41)	0.82 (±0.38)	0.82 (±0.59)	0.46 (±0.20)	2.34 (± 1.25)
New	5	1	—	0.84 (±0.35)	0.17	—	1.18 (±0.45)	0.70 (±0.43)	0.89 (±0.40)	0.59 (±0.33)	0.65 (±0.29)	2.40 (±1.51)
New	5	3	—	0.87 (±0.40)	0.10	0.33	1.33 (±0.51)	0.69 (±0.48)	0.87 (±0.39)	0.77 (±0.57)	0.71 (±0.27)	2.30 (±1.34)
New	40	1	—	0.99 (±0.41)	0.15	—	1.30 (±0.44)	0.87(±0.54)	1.15 (±0.48)	0.54 (±0.41)	0.96 (±0.35)	2.77 (±2.05)
Dimer	5	1	—	1.00 (±0.50)	0.14	—	1.63 (±1.09)	0.75 (±0.46)	1.18 (±0.46)	1.35 (±1.20)	0.77 (±0.30)	2.68 (±1.43)
Dimer	5	3	—	1.10 (±0.48)	0.16	0.28	1.69 (±1.01)	0.86 (±0.48)	1.25 (±0.44)	1.31 (±1.09)	0.89 (±0.38)	2.64 (±1.19)
Dimer	40	1	—	0.87 (±0.33)	0.43	—	0.96 (±0.80)	0.83 (±0.35)	1.20 (±0.47)	1.39 (±1.48)	1.05 (±0.65)	1.59 (±1.36)
Dimer	100	1	—	0.75 (±0.34)	0.37	—	0.88 (±0.68)	0.70 (±0.41)	1.18 (±0.43)	1.33 (±0.96)	1.09 (±0.64)	1.39 (±1.50)

^a^Initial protein structure used for FEP calculations. ^b^Simulation time for each λ window. ^c^Number of repeats. ^d^Mean unsigned error in kcal/mol compared to experiment after mean-centred fitting, numbers in parentheses are 99% confidence interval. ^e^Standard deviation in MUE if repeat calculations were performed. ^f^The new structure is available in the PDB with accession code 6EZF.

This favourable FEP result inspired us to further consider the endogenous state of FL PDE2 by building a dimeric form of the catalytic domains. Neither monomer could adopt an open H-loop conformation due to clashes with the other monomer, but maintaining one monomer in the inactive state, with H-loop in the active site, and using our new structure with an intermediate H-loop conformation allowed placing ligands in one binding site of the dimer. We used this model structure to perform initial classic MD simulations (to stabilize the system) prior to FEP. The 5 ns FEP protocol gave good results, ΔG MUE 1.00 ± 0.50 kcal/mol. Furthermore, improvements were seen with increased sampling, with 40 ns and 100 ns λ windows showing ΔG MUE of 0.87 ± 0.33 and 0.75 ± 0.34 kcal/mol respectively. The improvement was likely due to better equilibration of the model with time. Overall, the model showed good protein RMSD and RMSF for different perturbations, Figure S12, with RMSD typically in the range of 2–3 Å across the entire simulation time. Curiously, improvements were now seen for the small-to-large transitions as the MUE dropped to 1.39 ± 1.50 kcal/mol. Figure 3 shows how the ΔG and ΔΔG correlation with experiment is partially improved, bringing the ΔG prediction for the small molecules into a similar and more accurate energy range compared to the large compounds.

## Discussion and Conclusion

We present an interesting data set where FEP approaches have the potential to outperform classical static structure-based methods, because of the subtle changes in local protein conformation and pocket water network as the substituent is varied. We reported a small FEP study on a similar series of PDE2 inhibitors several years ago^25^ and also saw a large under predicted activity of a small compound containing only hydrogen as the equivalent **R**-group. Here we set out to understand this in more detail and used a larger set of new inhibitors. Logically, we started the FEP calculations using the crystal structure solved with ligands of the same chemical series. We quickly identified that with either of these structures an overall error in predicted ΔG in the range of 1.2 to 1.4 kcal/mol was the norm, and again the small molecules were worse predicted. This originated from the very large (ΔΔG > 3 kcal/mol) errors of the small-to-large perturbations. On the other hand, the small-small and large-large perturbations had acceptable errors around 1 kcal/mol.

We considered that the critical perturbations were simply too large and that protein and water rearrangements converged slowly. A variety of approaches was used to enhance sampling (length and number of λ windows, averaging of results, increasing REST region) but none resulted in significant improvement. Improving the ‘physical reality’ of the system by enforcing the presence of the waters needed to occupy the top-pocket for the small molecules also did not improve results. Small-to-small and large-to-large transitions showed good comparison with experiment. In contrast to our initial concerns, local sampling and movement of Leu770 was not a source of error. There were no beneficial effects of adding Leu770 to the REST region and we observed that solvent can enter the pocket if needed. Therefore, either solvent enters and the system rapidly adopts physical reality, or by cancellation of errors between very similar states, the small-to-small transitions performed well. Meanwhile the large-to-large transitions could be modelled in a physically realistic way and in general delivered ΔΔG MUE compared with experiment in a good range <1 kcal/mol. For the perturbations between the small and large molecules errors could arise from different sources and no longer cancel.

**Figure 4:**
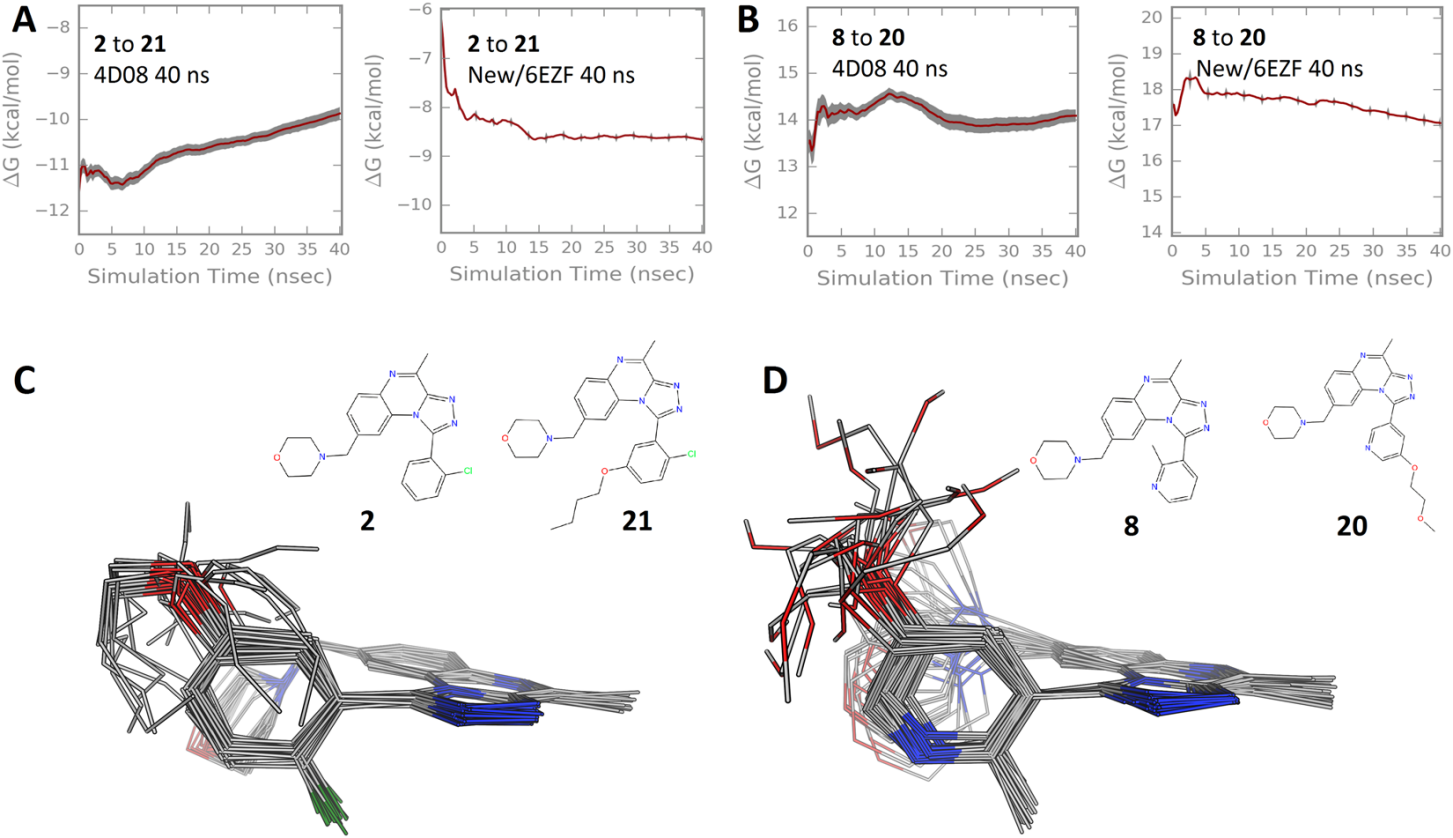
Details of small-to-large perturbations. (**A**) The change in free energy in the protein complex for the perturbation of **2** to **21** using different starting structures, 4D08 or New/6EZF. (**B**) The same as panel A but for perturbation of molecule **8** to **20**. The plots reveal the extent of convergence of the free energy change vs simulation time and that perturbation of **2** to **21** was improved with the new/6EZF structure. (**C**) For the worst performing small-to-large perturbation, the overlay of the 3D hybrid topology ligands (i.e. including dummy atoms) from the simulation of the first λ window. The structures are from the perturbation of **2** to **21** using the 4D08 structure and a 5 ns λ window protocol, the ΔΔG MUE compared to experiment was 5.16 kcal/mol (Bennet error 0.15 kcal/mol). (**D**) The same as C but for the best performing perturbation, **8** to **20** using the new/6EZF structure and a 40 ns λ window protocol. The ΔΔG MUE compared to experiment was 0.08 kcal/mol (Bennet error 0.19 kcal/mol).

What were the reasons for the error in predicting the small-to-large perturbations? Using the intermediate H-loop and especially the dimer model gave improvements in the overall ΔG and ΔΔG results, and even the ΔΔG for small-to-large changes. For some of the small-to-large perturbations with the 4D08 starting structure the free energy change in the complex was not converged even after 40 ns λ window simulations, Figs 4 and S13. This was not the case for the same perturbation with the new/6EZF crystal structure. Lack of convergence arises from insufficient overlap of states between λ windows, suggesting exploration of different conformational space. The ligand RMSDs were typically in a good range (<2–3 Å) and we had dismissed issues of excessive ligand movement. However, the simulation trajectories revealed that for some cases the larger molecules did not maintain their expected interactions in the hydrophobic top-pocket. This contrasts with the dimer simulations for example where the chain stayed in the top-pocket and made consistent interactions. The simulation interaction diagrams in supplementary information Figure S14 show how the large substituent chain could behave differently depending on the protein structure that was used.

This problem of exit of the larger molecules from the top-pocket confounded the convergence for small to large molecule transformations. Furthermore, the small-to-large perturbations require a linear chain of multiple (≥4) atoms that couple with the environment through the λ stages. In the first λ windows the ‘appearing’ atoms are represented as dummies with low van der Waals terms. Figure 4C and D shows the overlay of an evenly sampled 20 structures from the first λ window of a bad and good performing small-to-large perturbation. In the poor performing perturbation (MUE of 5.16 kcal/mol) the coupling atoms collapse out of the expected top-pocket binding pose, whilst for the better example (MUE 0.08 kcal/mol) more conformations remain. The poor performing perturbation does not maintain the similar conformation through the FEP λ steps meaning worse overlap and convergence.

Our main aim with this study is to provide a realistic dataset where local and large-scale protein motions are important. Such systems should be ideally suited for molecular dynamics based approaches, and although FEP performed better than standard approaches, it still struggled especially in the initial calculation attempts. When comparing results from different methods and protocols an important aspect is the statistical significance between differences in affinity predictions. We have provided all calculated results with 99% confidence intervals showing that small changes/improvements in MUE are not significant. Despite many attempts, no significant differences were seen for the modifications to the FEP protocols in Table 2. When changing the starting protein conformation, the results in Table 3 began to improve. Using a 5 ns λ simulation FEP protocol the MUE for ΔG of the new/6EZF structure was 0.84 ± 0.35 kcal/mol, and the MUE for the best FEP result was 0.75 ± 0.34 kcal/mol. Compared to the initial calculation (4D08 5 ns MUE of 1.34 ± 0.54 kcal/mol) the respective p-values were 0.051 and 0.023. FEP results were significantly better compared to other methods. Docking showed inverse correlation with experiment and comparison of MUE with the best MM/GBSA results was significant with a p-value of 0.0004. When focusing on the small set of outliers, small-to-large perturbations, an improvement in ΔΔG for just two or three perturbations is sufficient to make a difference to the overall mean MUE. Detailed inspection allows a physical rationalisation of the origin of the errors and the improvements in ΔΔG MUE were in some cases as large as >2 kcal/mol. Overall, this provides confidence in the presented analyses.

In summary, we set out to predict the activity of a series of PDE2 inhibitors using FEP. Small molecules were potent inhibitors (**2** pIC_50_ 8.57) and large molecules were also highly active (**4** pIC_50_ 8.14). Intermediate compounds showed a mix of affinity (**15** pIC_50_ 8.75 vs **16** pIC_50_ 6.42). The ΔG was accurately predicted for the molecules within the two sets (small and large). However, when all were combined the affinity of the small molecules was underestimated meaning it would have been difficult to apply FEP in a prospective manner. The inaccuracies arose from the perturbations between small-to-large molecules. The movement of H12 allowed large **R**-groups to depart from the top-pocket as did the coupling atoms in small-to-large perturbations. The instability of the coupling atoms prevents good predictions of the difference in ΔG of the small and large sets of molecules. This instability would normally not be seen because most chemical groups have internal constraints to maintain a similar 3D and chemical structure but this is a challenge for extended simple aliphatic chains. In the FL dimeric state of PDE2 the catalytic domains interact with each other likely blocking the H12 movement. Simulations performed with the new/6EZF structure did not show H12 opening and large ligands maintained their binding mode in the top-pocket as well as the decoupled region in some small-to-large perturbations. Hence, ΔΔG for small-to-large molecules improved leading to an overall improvement in the prediction of ΔG for both small and large molecules. Hence, this case study demonstrates the sensitivity of FEP results to the protein conformation, and surprisingly, that the typical ‘first-choice’ crystal structure may not be best. Overall, FEP approaches and technology are advancing^12,35^, and new datasets are needed to continue improving FE prediction methods^36^. We expect this study will be useful for future exploration.

## Methods

### X-ray crystallography

We report a new X-ray crystal structure of the catalytic domain of PDE2A amino acids Ser578 to Glu919. From cloning, residues MetHisAla were added at the N-terminus and LeuGlu as well as a His6-tag at the C-terminus. Protein was produced by expressing PDE2A construct in E. coli and subsequent purification using Ni-NTA affinity and gel filtration chromatography yielded homogenous protein with a purity of greater than 95% (coomassie stained SDS-PAGE) in preparative amounts. The purified protein was crystallized from 25% (w/v) PEG4000, 0.1 M Tris/HCl pH 8.25 and 0.2 M LiSO_4_ at 298 K by sitting-drop vapor diffusion. Prior to flash-freezing in liquid nitrogen the crystal was briefly immersed in the reservoir solution supplemented with 25% ethylene glycol and measured at a temperature of 100 K. The X-ray diffraction data were collected from complex crystals of PDE2A with the ligand **5** at the SWISS LIGHT SOURCE (SLS, Villigen, Switzerland) using cryogenic conditions. The crystals belong to space group P 21 21 21. Data were processed using the software XDS and XSCALE. Further details on data collection are in Table S1. Subsequent model building and refinement was performed with the software packages CCP4 and COOT, and again further details are found in supplementary information and Table S2. The ligand parameterisation and generation of the corresponding library files were carried out with CORINA.

### Protein/ligand preparation

All structures were prepared for computational work firstly using the “Protein Preparation Wizard” tool in Maestro with default settings, missing sidechains/atoms were fixed, any missing loops were modelled, protein protonation states were assigned with PROPKA at pH 7.0, binding site metals were retained and zero bond order constraints to neighbouring atoms were assigned, the hydrogen bonding network was optimized, ligand charges assigned, and a brief minimisation to RMSD 0.5 Å was performed. The dimeric model was built using the catalytic domain of the near FL crystal structure PDB 3IBJ as a template. One of the monomers was replaced with the New/6EZF structure that presents an intermediate H-loop conformation permitting a ligand to be bound in the active site. The second monomer was taken from the inactive state of 3IBJ. The new intermediate H-loop was simply placed by protein superposition with one of the catalytic domains of 3IBJ. Minimisation and refinement was performed to remove local clashes prior to further standard equilibration protocols.

Given the very high similarity for the ligands they were manually built using the crystalized ligands in 4D08 and 4D09 as template. The 21 molecules were prepared using the LigPrep tool in Maestro. The same ligand conformations and neutral ionisation state were used in all FEP protocols with the different protein structures. The ligands with larger R-groups clashed with the closed Leu770 conformation from PDB 4D09. These clashes were not resolved prior to the FEP calculations to understand if this could be accommodated during the FEP calculation without a priori knowledge of the Leu770 movement. For the alternative H-loop conformation crystal structure and dimer model, the ligands were placed by superposing the entire system onto the input for the PDB 4D08 calculations.

For analysis purposes, the compounds were divided into two subsets, small and large. The small subset fit in the closed Leu770 protein structure without any need for Leu770 movement, while the large subset requires the open Leu770 conformation to bind without clashes. The small compounds were: **2**, **6**, **7**, **8**, **9**, and **10**, and the large compounds were: **4**, **11**, **12**, **13**, **14**, **15**, **16**, **17**, **18**, **19**, **20**, **21**, **22**, **23** and **24**. Consequently, the types of perturbation were small-to-small, large-to-large, and small-to-large R-group.

### Free energy perturbation procedure

FEP calculations were performed using v2017-1 of the Schrödinger modeling suite. The OPLSv3 force field was used, GPU-enabled parallel molecular dynamics (MD) engine Desmond v3.9, the REST enhanced sampling technique, and the cycle-closure correction algorithm to generate free energy estimates^12^. Unless otherwise stated, the REST region only included heavy atoms of the ligand. The aligned ligands in the protein binding site were passed to the FEP mapper to automatically define the perturbation network. Three missing force field torsion parameters were added by additional QM calculations and fitting using the ffbuilder module. These corresponded to the bond between the triazo and phenyl rings in molecules **2** (and others) and **7**, as well as the CH_2_-CH_2_F dihedral in molecule **19**. We report theoretical error estimates based on standard deviation of repeat simulations, and compare with experiment via the correlation coefficient (R^2^) and the mean unsigned error (MUE). MUEs are also reported with 99% confidence intervals.

### Data Availability Statement

The datasets generated during and/or analysed during the current study are available from the corresponding author on reasonable request. Input structures can be found at the following link. https://drive.google.com/file/d/10O2nk-Gk4-MypWLYESS2Wpgl5wxBWZSL/view?usp=sharing. The PDE2 crystal structure is available in the PDB with accession code 6EZF.

## Electronic supplementary material

Supporting information
