# Supplementary material for: Predicting Binding Free Energies of PDE2 Inhibitors. The Difficulties of Protein Conformation
Source: Sci Rep. 2018 Mar 20;8:4883. doi: 10.1038/s41598-018-23039-5 (PMC5861043; doi:10.1038/s41598-018-23039-5)
Supplement: Supplementary file 1 — Supporting information [file 41598_2018_23039_MOESM1_ESM.pdf]

Supplementary Information:

Predicting Binding Free Energies of PDE2 Inhibitors. The Difficulties of Protein Conformation

Laura Perez-Benito[a,‡], Henrik Keränen[a,†,‡], Herman van Vlijmen[a], Gary Tresadern[a,]*

[a] Computational Chemistry, Janssen Research & Development, Janssen Pharmaceutica N. V., Turnhoutseweg 30, B-2340 Beerse, Belgium.

‡ These authors contributed equally.

* Corresponding author phone: +32 1464 1569. E-mail: gtresade@its.jnj.com

Present Address: † Computational Chemistry and Struct. Biol., H. Lundbeck A/S, Ottiliavej 9, 2500 Valby, Denmark.

Contents:

- Crystallography Methods
- Figure S1. Crystal structure of PDE2 catalytic domain structure solved with molecule **5**.
- Figure S2. Comparing H-loop conformations in published PDE2 crystal structures
- Figure S3. Mapper showing the 34 perturbations used for FEP calculations.
- Comparison with other methods
    - Table S3. Comparison of docking and MM/GBSA calculation results to experiment.
    - Figure S4. The correlation of predicted ΔG from docking and MM/GBSA calculations with experiment.
- Modifying mappers in the standard 4D08 and 4D09 FEP calculations
    - Figure S5. Modified larger mapper containing 79 perturbations between the 21 molecules.
    - Figure S6. Modified larger mapper containing no perturbations between small and large molecules that would create a chain of more than 3 dummy atoms.
    - Figure S7. Modified LOMAP mapper containing 25 perturbations.
- Figure S8. The dihedral angle of Leu770 during FEP MD simulations
- Figure S9. Opening movement of Helix 12 (H12) during MD simulations.
- Figure S10. Protein RMSD, and RMSF (root mean square fluctuation) for λ 0 and 1 states for selected perturbations from the 4D08 5 ns protocol.
- Figure S11. Protein RMSD, and RMSF (root mean square fluctuation) for λ 0 and 1 states for selected perturbations from the 4D08 40 ns protocol.
- Figure S12. Protein RMSD, and RMSF (root mean square fluctuation) for λ 0 and 1 states for selected perturbations from the dimer 100 ns protocol.
- Figure S13. Convergence of the free energy for selected perturbations in the 40 ns λ window simulations performed using the 4D08 starting structure.
- Figure S14. RMSD and ligand interaction diagrams summarizing MD trajectories for small-to-large perturbation of molecule **2** to **21**.

Input structures can be accessed at the following link:

https://drive.google.com/file/d/10O2nk-Gk4-MypWLYESS2Wpgl5wxBWZSL/view?usp=sharing

Crystallography Methods

Table S1. Data collection and processing statistics for reported PDE2A crystal structure

X-ray source	PXI/X06SA (SLS[1])
Wavelength [Å]	1.0000
Detector	PILATUS 6M
Temperature [K]	100
Space group	$P\ 2_1\ 2_1\ 2_1$
Cell: a; b; c; [Å]<br>α; β; γ; [°]	54.24; 65.32; 104.39<br>90.0; 90.0; 90.0
Resolution [Å]	1.50 (1.72-1.60)[2]
Unique reflections	59438 (9950)[2]
Multiplicity	7.0 (7.5)[2]
Completeness [%]	98.8 (100.0)[2]
R_{sym} [%]	4.7 (41.5)[2]
R_{meas} [%]	4 5.1 (44.5)[2]
Mean(I)/sd	21.23 (5.61)[2]

Method Structure Modelling and Refinement

The phase information necessary to determine and analyse the structure was obtained by molecular replacement. A previously solved structure of PDE2A was used as a search model. Subsequent model building and refinement was performed with the software packages CCP4 and COOT. For the calculation of the free R-factor, a measure to cross-validate the correctness of the final model, about 1.6 % of measured reflections were excluded from the refinement procedure (see Table S2). Anisotropic B-factor refinement (using REFMAC5, CCP4) was carried out,

which resulted in lower R-factors and higher quality of the electron density map. The ligand parameterization and generation of the corresponding library files were carried out with CORINA. The water model was built with the "Find waters"- algorithm of COOT by putting water molecules in peaks of the Fo-Fc map contoured at 3.0 σ followed by refinement with REFMAC5 and checking all waters with the validation tool of COOT. The criteria for the list of suspicious waters were: B-factor greater 80 $Å^2$, 2Fo-Fc map less than 1.2 σ, distance to closest contact less than 2.3 Å or more than 3.5 Å. The suspicious water molecules and those in the active site (distance to inhibitor less than 10 Å) were checked manually. The occupancy of side chains, which were in negative peaks in the Fo-Fc map (contoured at -3.0 σ), were set to zero and subsequently to 0.5 if a positive peak occurred after the next refinement cycle. The Ramachandran Plot analysis of the final model showed 93.1 % of all residues in the most favoured region, 6.9 % in the additionally allowed region, and 0.0 % in the generously allowed region. No residues were found in the disallowed region (Table S2). Statistics of the final structure and the refinement process are listed in Table S2.

Table S2. Refinement statistics for reported PDE2A crystal structure

Resolution [Å]	55.37-1.50
Number of reflections (working / test)	58498 / 940
R_{cryst} [%]	15.1
R_{free} [%]	19.8
Total number of atoms:	
Protein	3023
Water	351
Ligand	16
Glycerol	18
Zinc	1
Sulphate	5
Magnesium	1
Sodium	1
Deviation from ideal geometry:	
Bond lengths [Å]	0.008

Figure S1. Crystal structure of PDE2 catalytic domain structure solved with molecule **5**. (A) The catalytic domain with the H-loop (yellow) closed over the active site entrance, molecule **5** (magenta) is bound and amino acids Leu770 and Met847 (green) can adopt two conformations. (B) Close-up of the binding site with same color scheme as A.

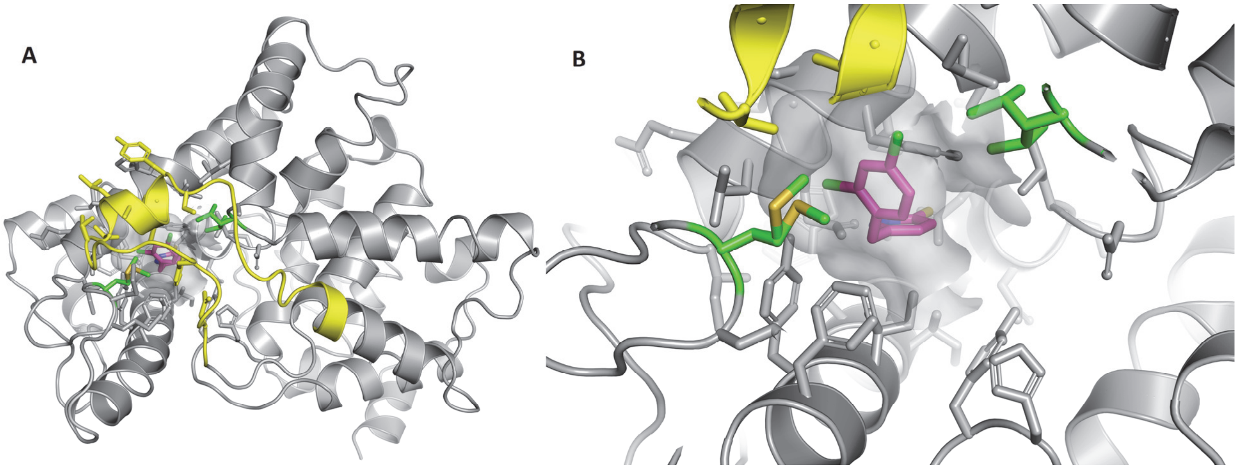

Figure S2. Comparing H-loop conformations in published PDE2 crystal structures. H-loop (702-728) colored yellow, M-loop (830-856) magenta, and ligands (green). Existing structures only show open (majority) or closed (inside active-site) H-loop conformations.

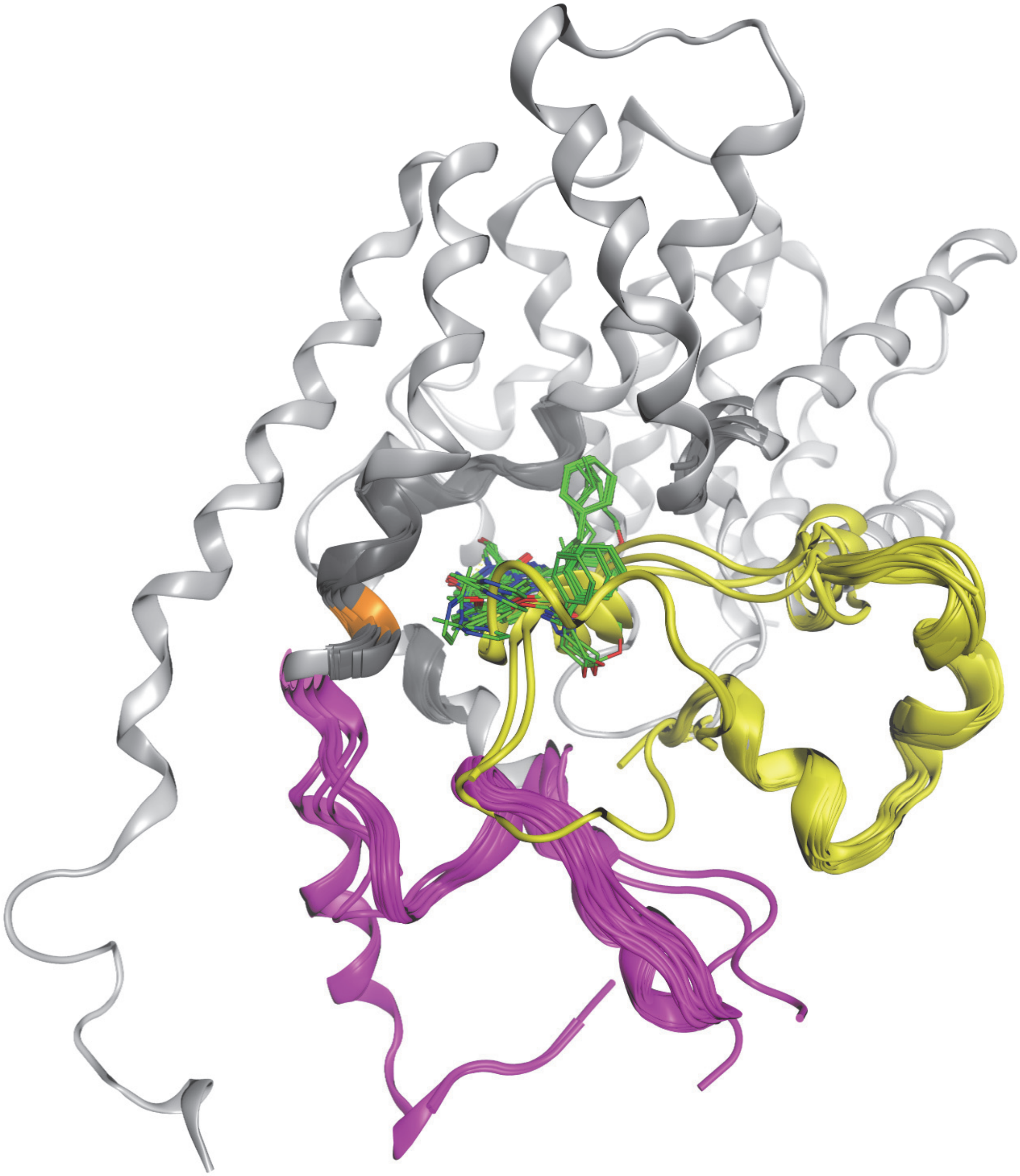

Figure S3. Mapper showing the 34 perturbations used for FEP calculations.

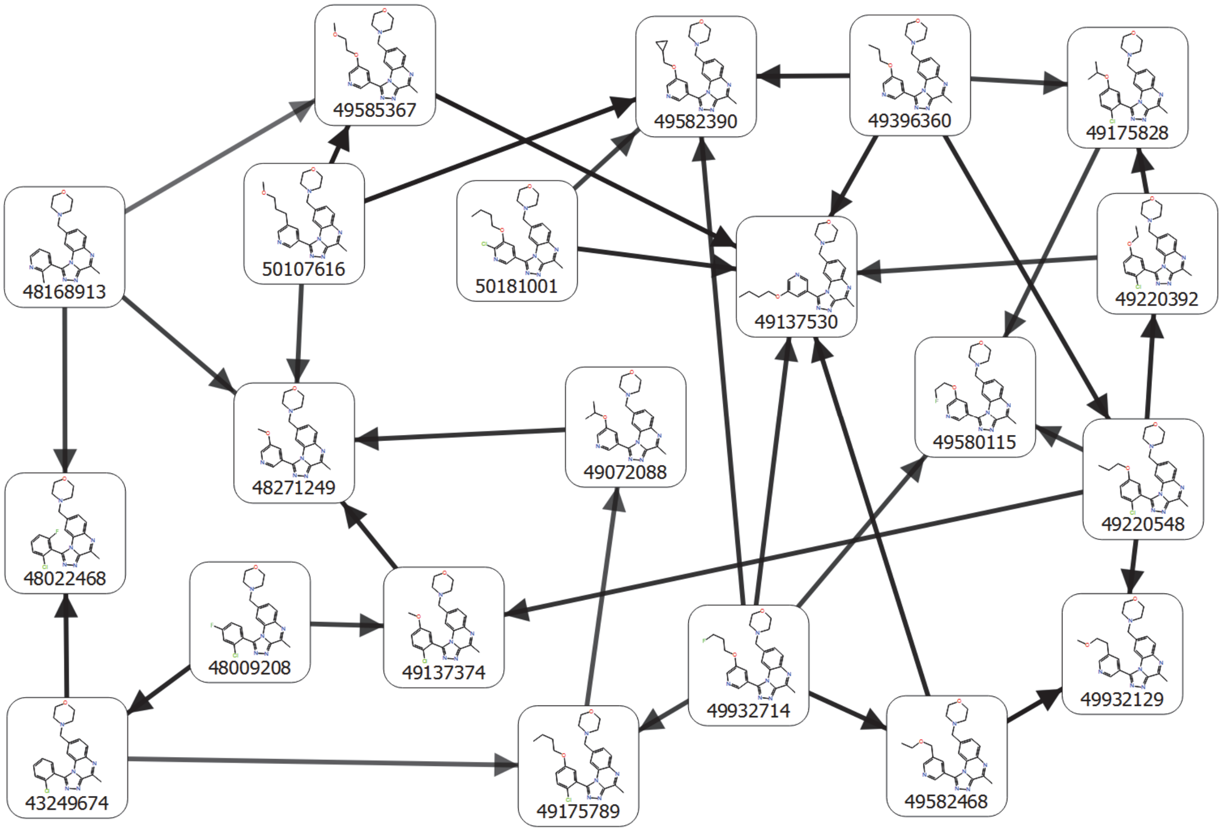

Text list of mutations in large mapper: 19 -> 4, 11 -> 4, 17 -> 4, 14 -> 4, 22 -> 4, 20 -> 4, 11 -> 15, 13 -> 11, 13 -> 18, 17 -> 18, 14 -> 15, 15 -> 12, 13 -> 9, 13 -> 12, 14 -> 13, 19 -> 24, 14 -> 24, 22 -> 24, 23 -> 24, 8 -> 10, 16 -> 10, 23 -> 10, 9 -> 10, 19 -> 21, 19 -> 12, 19 -> 17, 23 -> 20, 8 -> 20, 8 -> 24, 2 -> 24, 6 -> 2, 2 -> 21, 2 -> 9, 21 -> 16.

Comparison with other methods.

Method

For comparison of performance of FEP with other methods, Glide SP docking and MM-GBSA calculations were performed. Glide software (Release 2017-1) from Schrödinger was used. All PDE2 protein structures were prepared as described for the FEP calculations. For docking the crystalized ligand in each protein structure was used to place the grid box. Active site waters were retained. The 21 ligand molecules were prepared for docking using the LigPrep tool. Additional sampling was performed by using the expanded sampling option and up to 15 initial poses were passed to post-docking minimization. All other docking parameters were set to the defaults.

The MM-GBSA calculations were performed using the same inputs for the FEP calculations. The VSGB solvation model was used along with force field minimization of the ligand. Results are reported from two approaches: a default that performs no protein minimization and a second approach that minimizes an 8 Å radius of amino acids surrounding the binding site (using the same active region for all ligands). All results were scaled/centered to the mean of the experimental activity, analogous to the FEP calculations.

Results

Results are summarized in Table 1 and Figure SX shown below. Docking with 4D08 or the New/6EZF crystal structures showed an anticorrelation with the experimental affinities. Meanwhile, using the 4D09 structure, with the closed Leu770 conformation, four of the large compounds could not be docked, and hence the approach is inadequate. Meanwhile the MM/GBSA calculations predicted a very large spread in the calculated ΔG and no correlation. Some large molecules also failed when performing the static MM/GBSA approach with the closed 4D09 structure. Overall, given the very large MUE and lack of any correlation, neither docking nor MM/GBSA methods are useful for this dataset.

Table S3. Comparison of docking and MM/GBSA calculation results to experiment.

	ΔG (kcal/mol)			
Starting Structure	MUE	R^2	Slope	Comment
Docking				
4D09 [a]	0.80 (± 0.33)	0.00	0.02	Multiple compounds failed
4D08	1.21 (± 0.40)	0.50	-0.52	Inverse correlation
New/6EZF	1.19 (± 0.45)	0.35	-0.50	Inverse correlation
MM/GBSA static				
4D09 [a]	20.48 (± 10.46)	0.07	10.32	Multiple compounds failed
4D08	6.94 (± 3.74)	0.08	3.18	
New/6EZF	15.88 (± 8.86)	0.02	4.02	
MM/GBSA minimization				
4D09	9.39 (± 4.94)	0.02	2.12	
4D08	13.77 (± 4.61)	0.23	9.07	
New/6EZF	10.40 (± 4.45)	0.04	2.86	

[a] Due to closed Leu770 conformaton many compounds cannot dock. Properties only provided for compounds that could be successfully docked or which did not fail in the static MM/GBSA calculations.

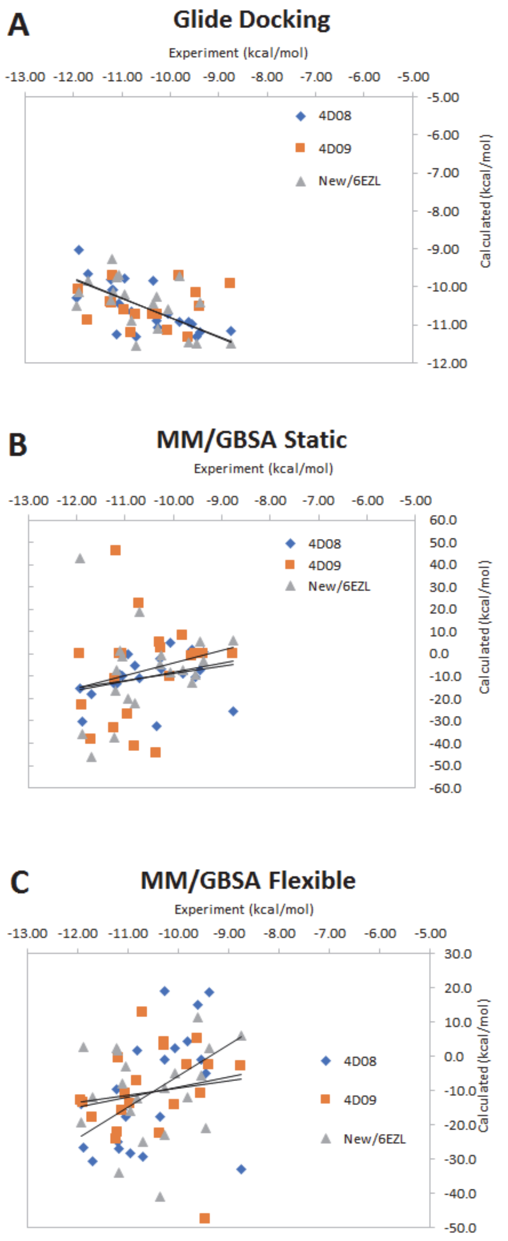

Figure S4. The correlation of predicted ΔG from docking and MM/GBSA calculations with experiment. Panel A docking, B MM/GBSA with static receptor, C MM/GBSA with minimization around the ligand.

Modifying mappers in the standard 4D08 and 4D09 FEP calculations

Increasing the mapper size

We investigated if a larger map would improve the predictions. The number of connections between compounds was increased from 34 to 79, see Figure S5. For the 4D08 starting structure the MUE for the ΔG was 1.35 ± 0.57 kcal/mol, and R^2 was 0.0.

Crystal structure	λ window (ns)	*n*	MUE (kcal/mol)	99% CI	Max MUE	Min MUE	R^2
4D08	5	1	1.35	0.57	3.15	0.01	0.00

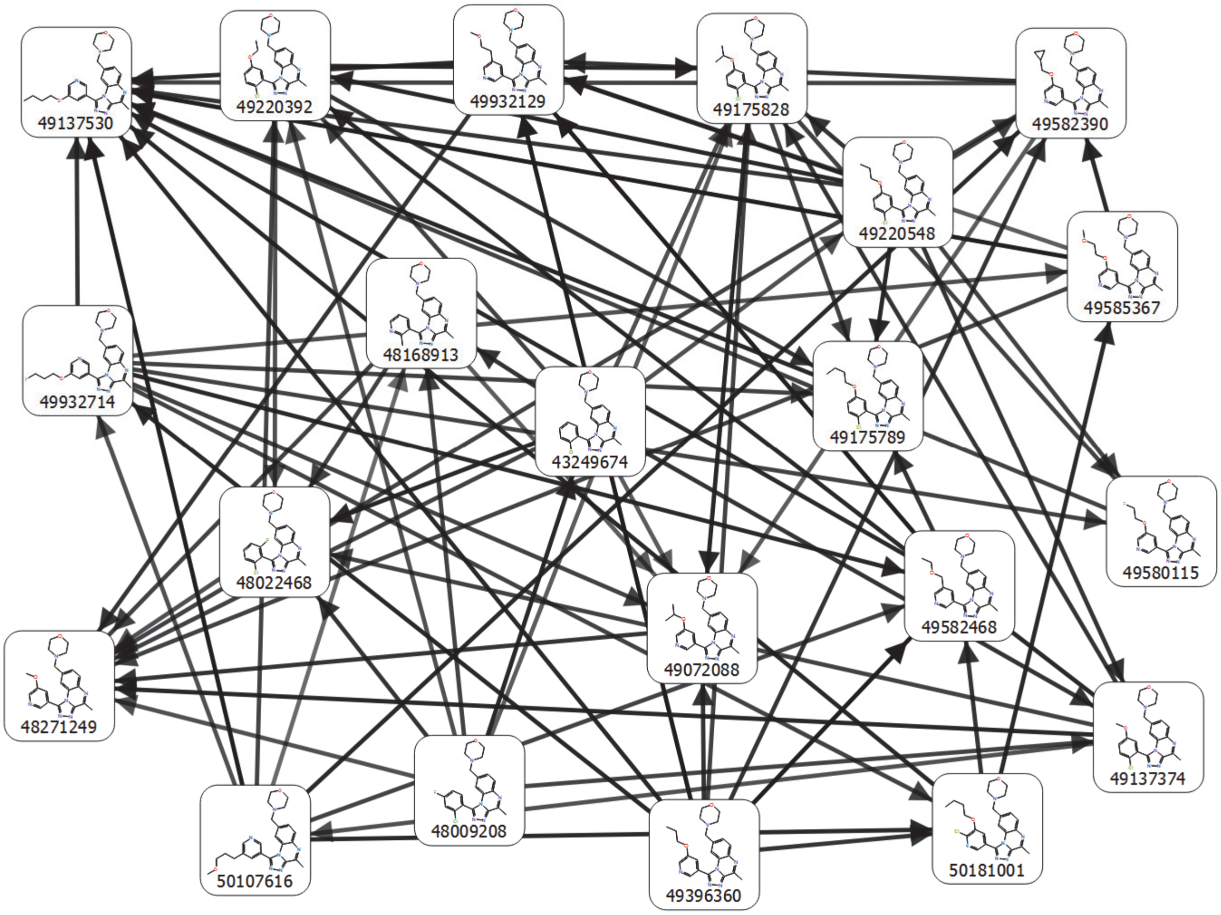

Figure S5. Modified larger mapper containing 79 perturbations between the 21 molecules. Text list of mutations in large mapper: 19 -> 4, 18 -> 4, 13 -> 4, 24 -> 4, 23 -> 4, 21 -> 4, 12 -> 4, 17 -> 4, 14 -> 4, 22 -> 4, 20 -> 4, 11 -> 15, 2 -> 11, 9 -> 11, 11 -> 24, 11 -> 21, 6 -> 11, 23 -> 11, 13 -> 11, 13 -> 18, 24 -> 18, 18 -> 10, 17 -> 18, 14 -> 18, 20 -> 18, 2 -> 15, 9 -> 15, 15 -> 21, 15 -> 16, 15 -> 12, 6 -> 15, 14 -> 15, 13 -> 15, 13 -> 9, 13 -> 24, 2 -> 13, 13 -> 21, 13 -> 12, 24 -> 10, 20 -> 24, 24 -> 16, 14 -> 24, 23 -> 24, 9 -> 10, 8 -> 10, 24 -> 10, 2 -> 10, 16 -> 10, 6 -> 10, 20 -> 10, 19 -> 20, 19 -> 21, 19 -> 16, 19 -> 12, 19 -> 17, 14 -> 19, 19 -> 22, 23 -> 19, 9 -> 23, 23 -> 8, 23 -> 17, 23 -> 22, 8 -> 24, 2 -> 8, 8 -> 16, 6 -> 8, 9 -> 24, 2 -> 24, 6 -> 24, 2 -> 16, 6 -> 2, 2 -> 9, 14 -> 16, 17 -> 21, 14 -> 17, 22 -> 17, 6 -> 9, 14 -> 22, 22 -> 20.

Removing edges or molecules

We then studied the effect the different perturbations were having on the overall affinity predictions. New maps removed connections and compounds. The ΔG was recalculated to investigate the effect the different subsets were having on the overall predictions.

- Connections between the small-to-large were removed: Using the 4D09 starting structure the MUE for the overall ΔG was 1.64 ± 0.66 kcal/mol, for the small compounds 2.34 ± 1.30 kcal/mol, and for the large compounds 1.36 ± 0.71 kcal/mol. Using the 4D08 structure, the MUE for the overall ΔG was 1.41 ± 0.61 kcal/mol, for the small compounds was 2.34 ± 1.30 kcal/mol, and for the large 1.36 ± 0.71 kcal/mol.
- All large compounds were removed: The MUE for ΔG was 1.04 ± 0.49 kcal/mol for the 4D09 structure and 0.65 ± 0.51 kcal/mol for the 4D08 structure.
- All small compounds were removed: The MUE was 1.2 ± 0.53 kcal/mol for the 4D09 structure and 1.05 ± 0.61 kcal/mol for the 4D08 structure.
- We rebuilt an entirely new mapper, Figure S6, which avoided perturbations that created more than 3 linearly connected dummy atoms. This approach also failed to deliver improvements. Using the 4D08 structure, the MUE for the overall ΔG was 1.43 ± 0.58 kcal/mol, for the small compounds was 2.47 ± 0.65 kcal/mol, and for large compounds was 1.01 ± 0.56 kcal/mol.
- We used the LOMAP tool to recommend a perturbation mapper for the same set of 21 compounds. It suggested a 25-edge mapper, Figure S7, that was used to calculate a 5 ns λ window FEP protocol using the 4D08 structure. This approach also did not deliver improvements. the MUE for the overall ΔG was 1.33 ± 0.55 kcal/mol, for the small compounds was 2.26 ± 0.54 kcal/mol, and for large compounds was 0.96 ± 0.57 kcal/mol.
- Using the default 34 perturbation mapper the number of λ windows was doubled to 24 per perturbation. With the 4D08 structure, the MUE for the overall ΔG was 1.24 ± 0.56 kcal/mol,

for the small compounds was 2.12 ± 0.72 kcal/mol, and for the large compounds was 0.89 ± 0.59 kcal/mol.

Hence increasing lambda windows or removing the small-to-large perturbations does not impact the overall quality of prediction, but removing one of the two groups, either all small or large compounds improves the MUE prediction.

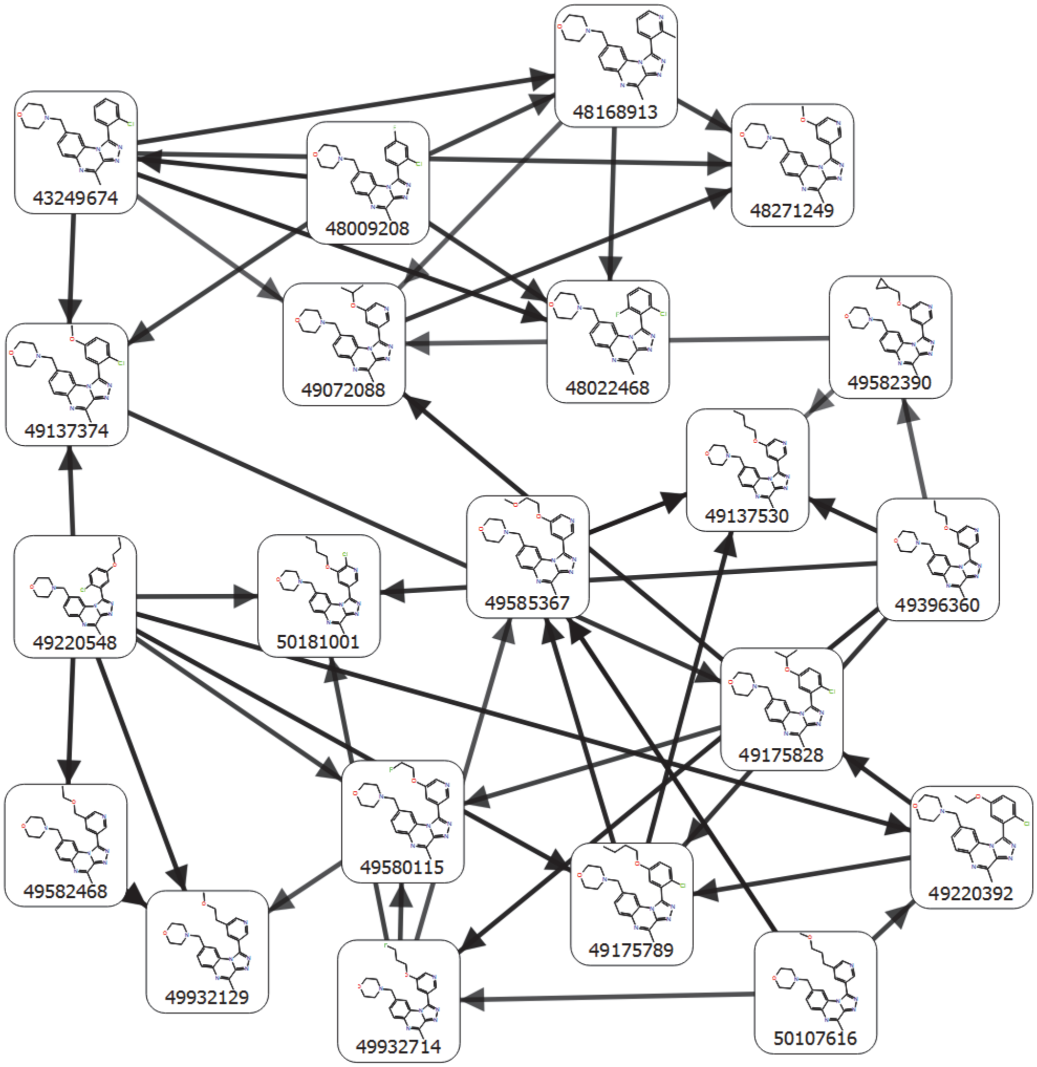

Figure S6. Modified larger mapper containing no perturbations between small and large molecules that would create a linear chain of more than 3 dummy atoms. Text list of mutations in large mapper: 24 -> 4, 14 -> 4, 20 -> 4, 21 -> 4, 11 -> 21, 11 -> 15, 13 -> 11, 23 -> 11, 13 -> 18, 12 -> 18, 17 -> 18, 9 -> 15, 15 -> 12, 15 -> 16, 13 -> 9, 13 -> 21, 13 -> 12, 13 -> 17, 13 -> 22, 14 -> 24, 24 -> 16, 8 -> 10, 2 -> 10, 16 -> 10, 14 -> 19, 19 -> 22, 19 -> 20, 19 -> 12, 23 -> 19, 23 -> 20, 6 -> 8, 2 -> 8, 8 -> 16, 8 -> 24, 6 -> 24, 2 -> 24, 2 -> 9, 2 -> 16, 6 -> 2, 14 -> 21, 21 -> 20, 6 -> 9, 14 -> 22.

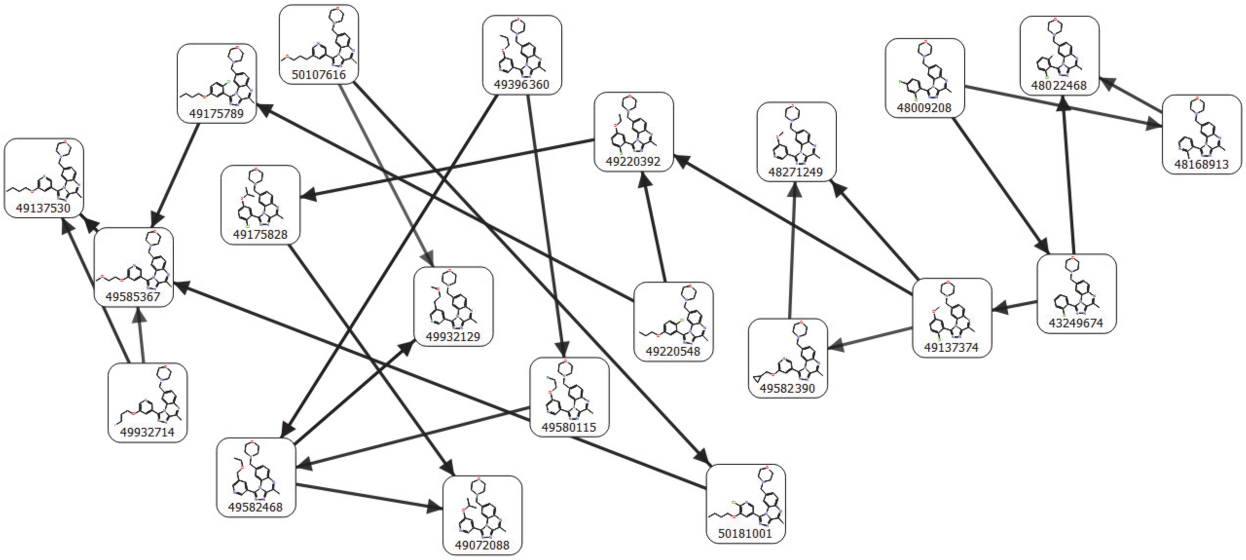

Figure S7. Modified LOMAP mapper containing 25 perturbations. This mapper was generated using the LOMAP tool which generally provides more conservative perturbations. Text list of mutations in mapper: 19 -> 4, 20 -> 4, 11 -> 15, 9 -> 11, 13 -> 11, 23 -> 18, 17 -> 18, 15 -> 16, 13 -> 21, 24 -> 10, 9 -> 24, 9 -> 10, 19 -> 20, 21 -> 20, 22 -> 20, 6 -> 8, 8 -> 7, 2 -> 7, 6 -> 2, 2 -> 9, 17 -> 16, 14 -> 17, 12 -> 17, 14 -> 12, 23 -> 22.

Figure S8. Comparing the dihedral angle ($\chi 1$) of Leu770 during FEP MD simulations for the perturbation between molecules **2** (small) and **21** (large) and using different starting protein structures. Angle from approx. -110° to 60° represents closed state. Regardless of whether starting with the open or closed state (PDBs 4D08 or 4D09 respectively) Leu770 shows similar behavior, although it was more often in an open conformation for simulations with the small molecule **2** than compared to the large molecule **21**.

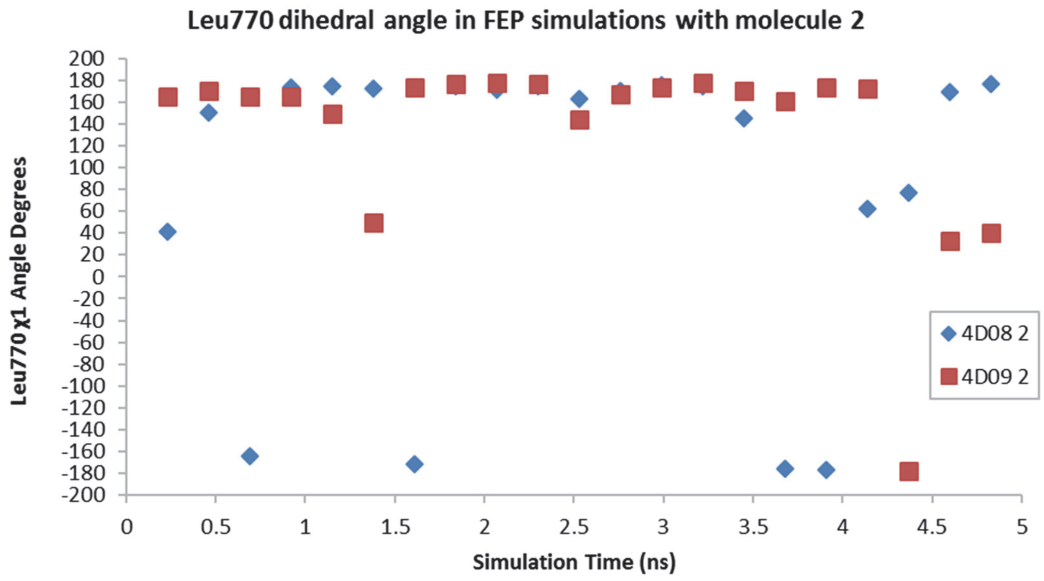

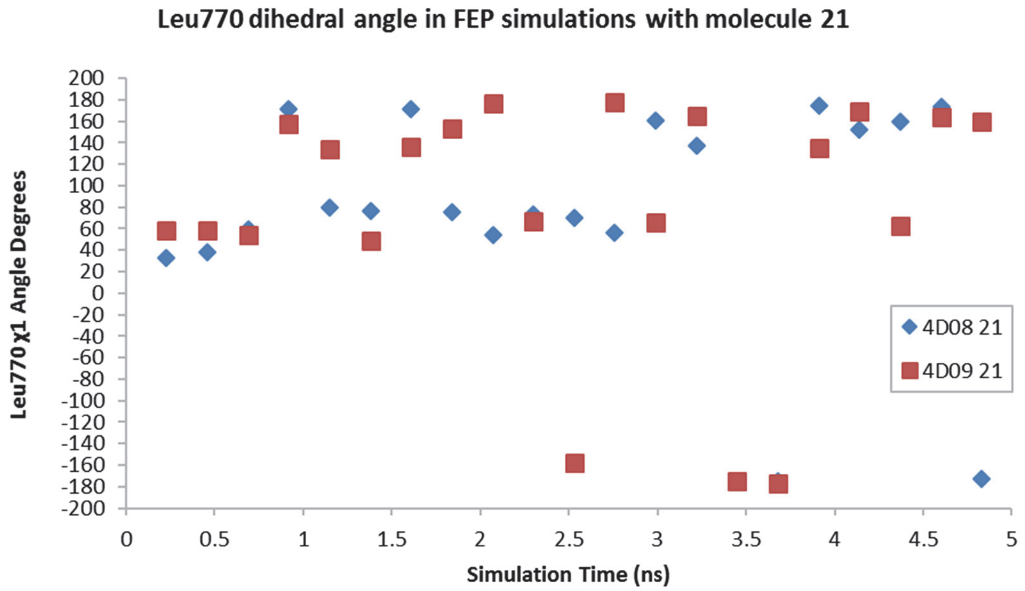

Figure S9. Opening movement of Helix 12 (H12) during MD simulations. The plot shows the distance between Leu770 and Thr805 during different MD simulations with molecules **2** and **21**, compared to the distance in X-ray structure. Simulations were performed with different starting structures, only one example shown using the new/6EZF crystal structure was capable of maintaining H12 in its expected closed orientation.

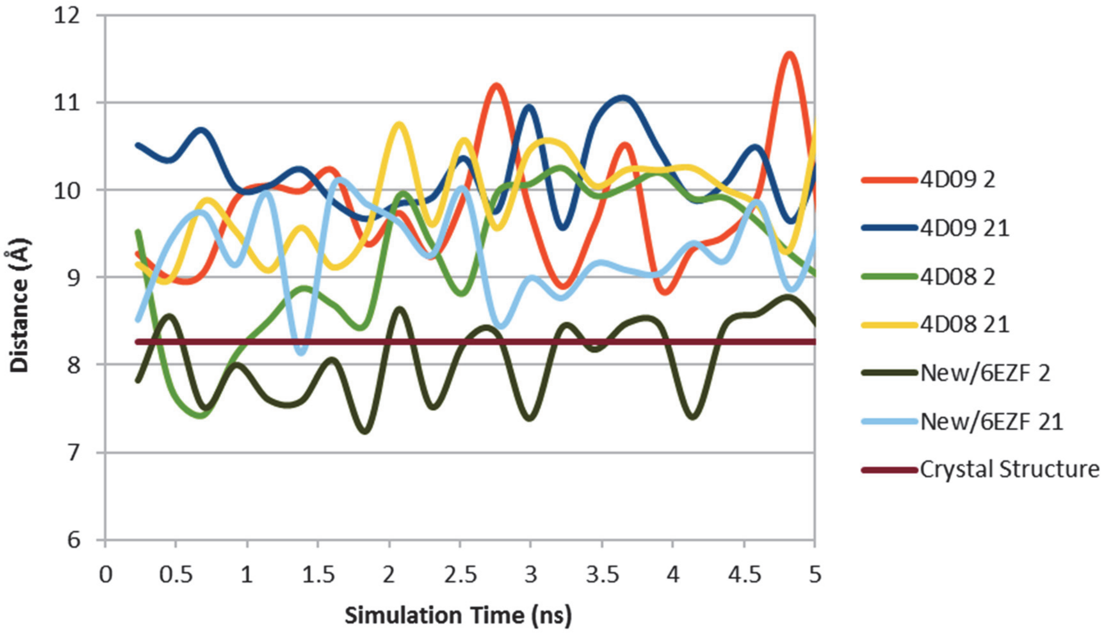

Figure S10. Protein RMSD, and RMSF (root mean square fluctuation) for λ 0 and 1 states for selected perturbations. Plots from 5 ns λ window simulations performed using the 4D08 crystal structure. In the RMSF plot, Leu770 is amino acid #195 and H12 extends for a further ~20 amino acids, amino acids that interact with the ligand have vertical bars.

Perturbation | Protein RMSD | Protein RMSF per amino acid

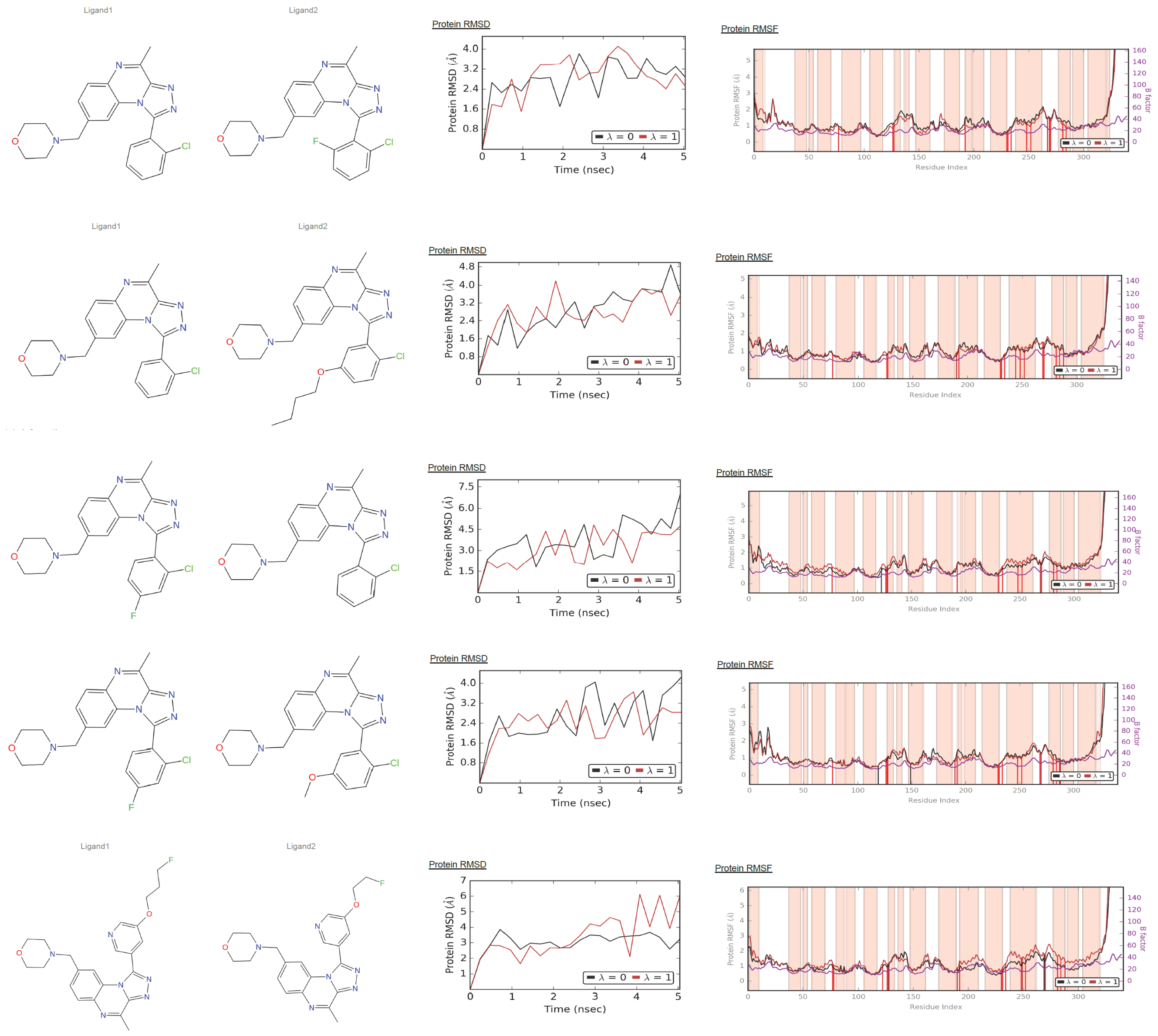

Figure S11. Protein RMSD, and RMSF (root mean square fluctuation) for λ 0 and 1 states for selected perturbations. Plots from 40 ns λ window simulations performed using the 4D08 crystal structure. The residue index for Leu770 is amino acid #195 and H12 extends for a further ~20 amino acids.

Perturbation | Protein RMSD | Protein RMSF per amino acid

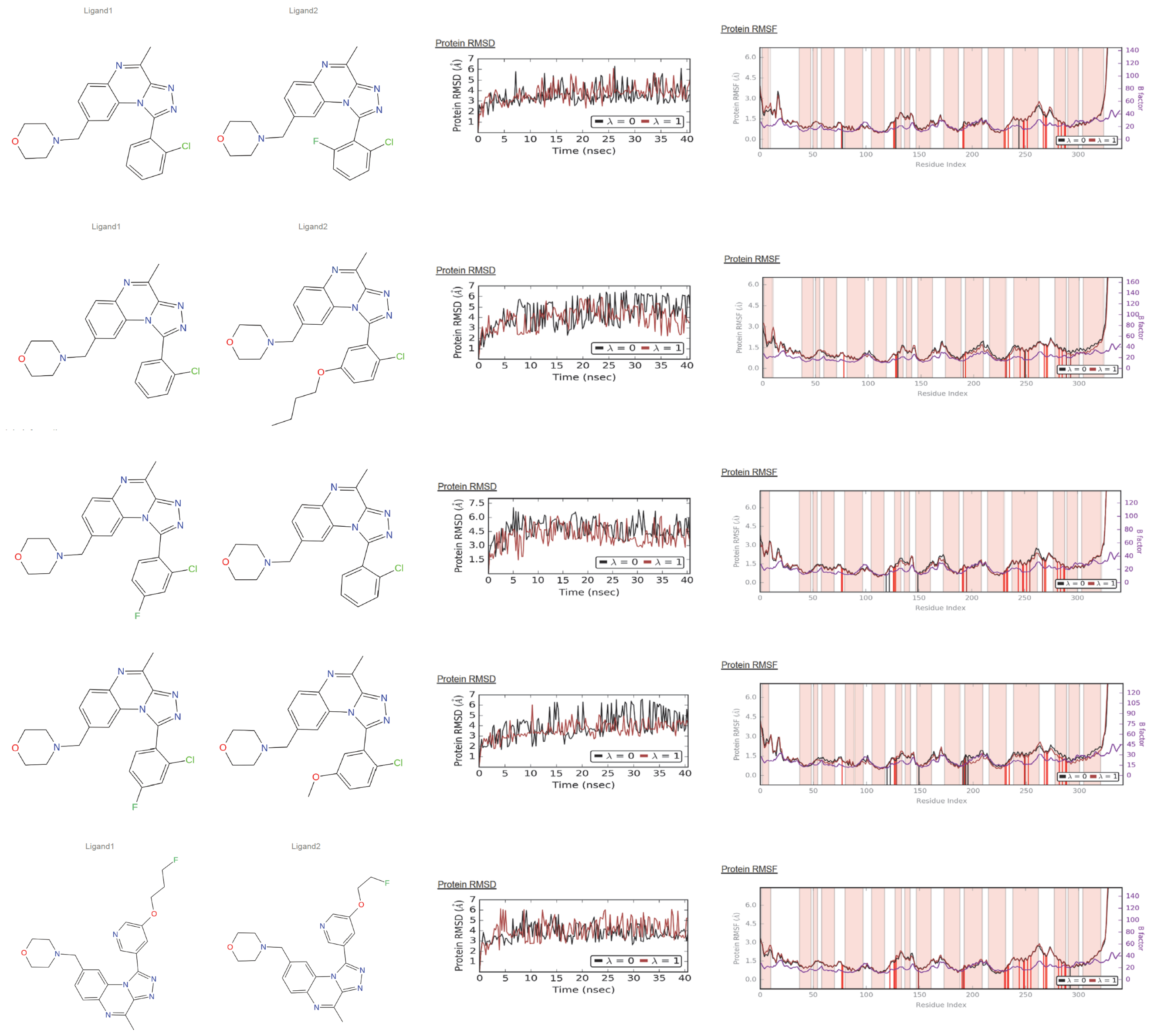

Figure S12. Protein RMSD, and RMSF (root mean square fluctuation) for λ 0 and 1 states for selected perturbations. Plots from 40 ns λ window simulations performed using the dimer model structure. The residue index for Leu770 is amino acid #195 and H12 extends for a further ~20 amino acids.

Perturbation Protein RMSD Protein RMSF per amino acid

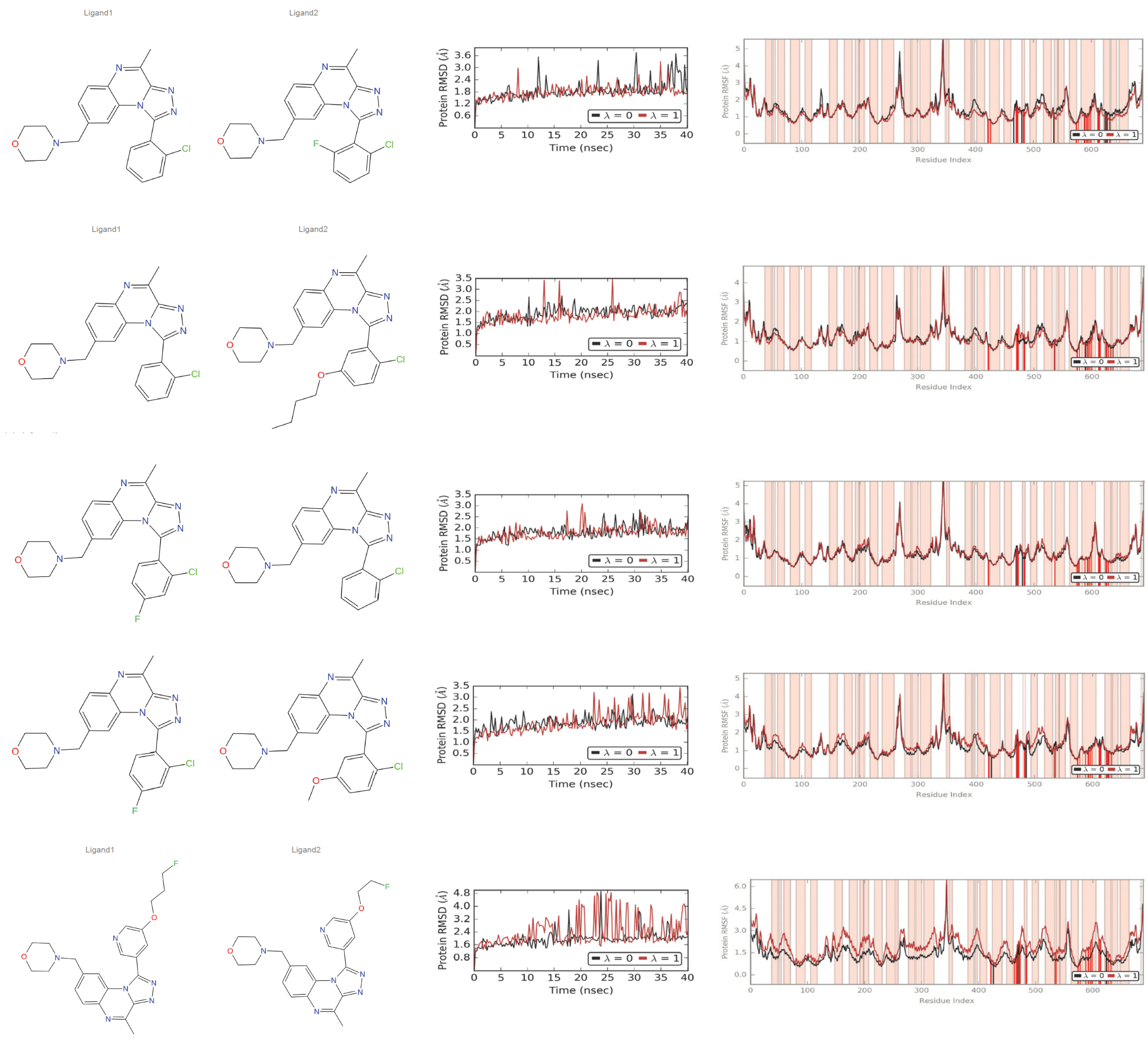

Figure S13. Convergence of the free energy for selected perturbations in the 40 ns λ window simulations performed using the 4D08 starting structure.

Perturbation | Free energy convergence for the complex leg

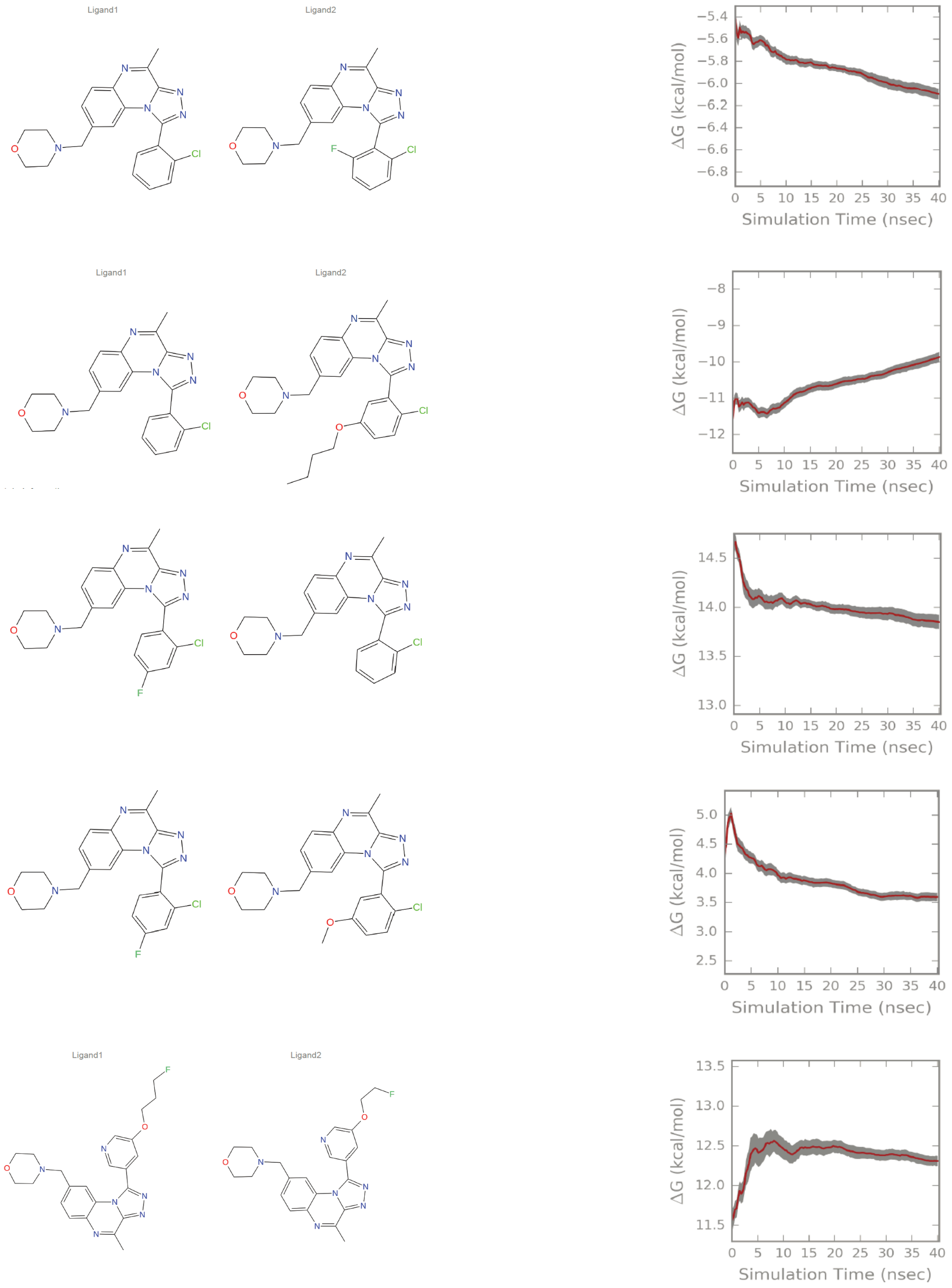

Figure S13 continued. Convergence of the free energy for selected perturbations in the 40 ns λ window simulations performed using the New/6EZF starting structure.

Perturbation | Free energy convergence for the complex leg

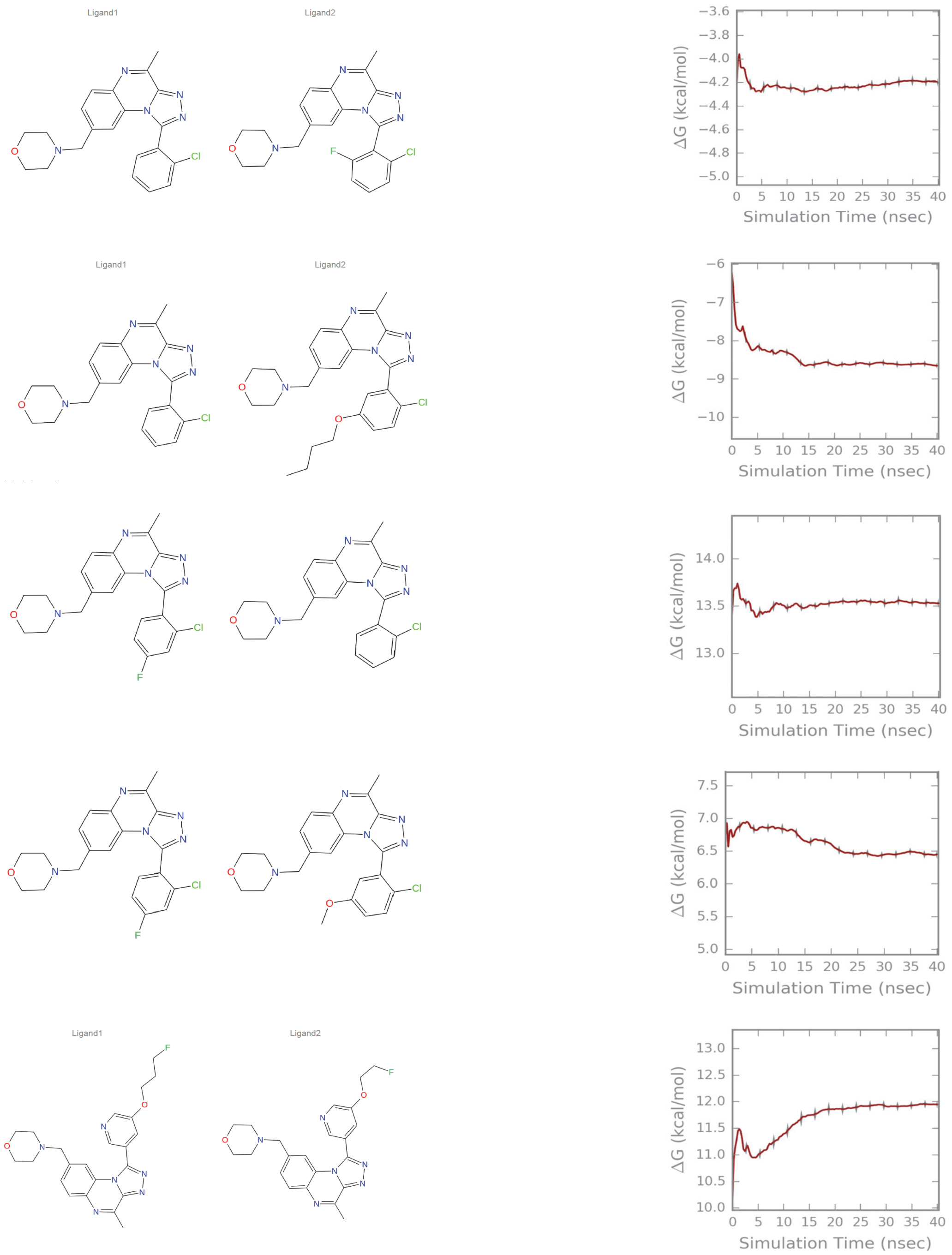

Figure S14. RMSD and ligand interaction diagrams summarizing MD trajectories for small-to-large perturbation of molecule **2** to **21**.

Perturbation & FEP protocol	RMSD ligands	Convergence	Simulation interaction diagram	
			Ligand 1	Ligand 2
**2-21**<br>4D08 5 ns	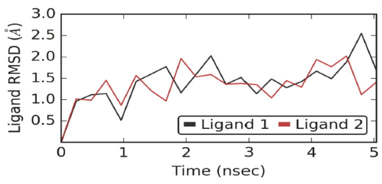	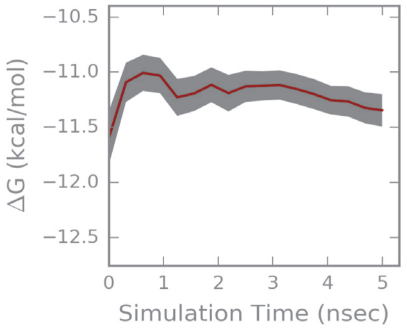	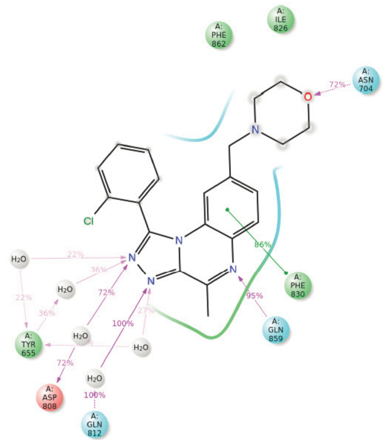	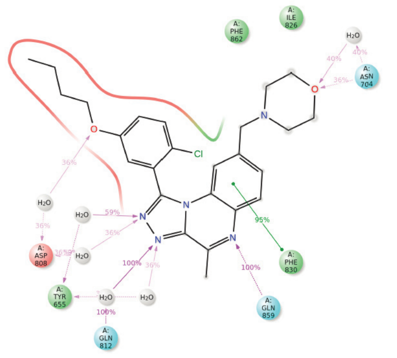
**2-21**<br>4D08 40 ns	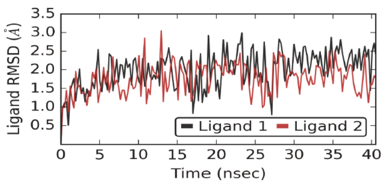	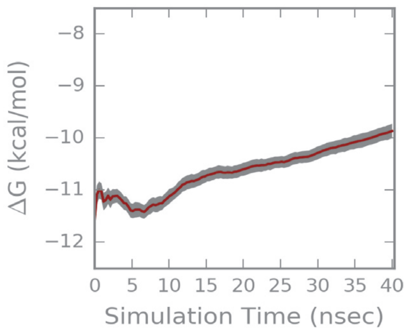	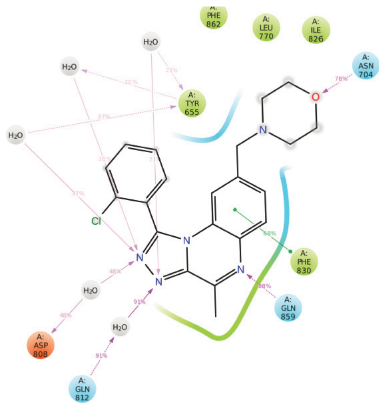	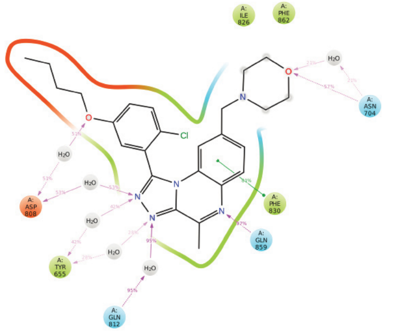

**2-21** New/6EZF 40 ns	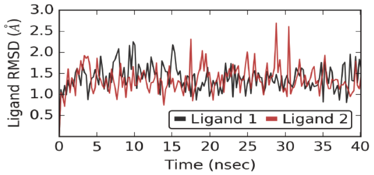	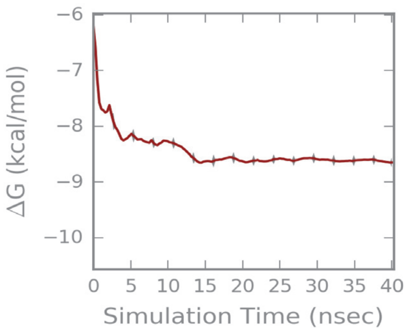	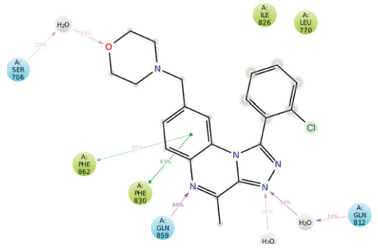	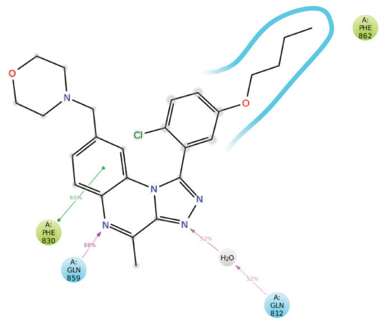
**2-21** Dimer 40 ns	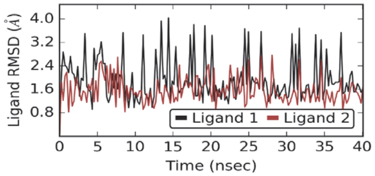	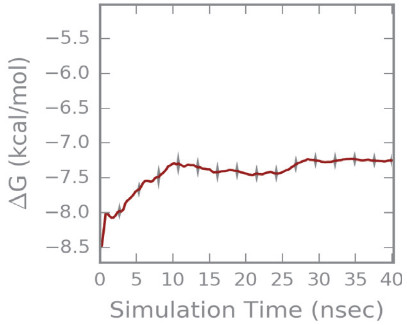	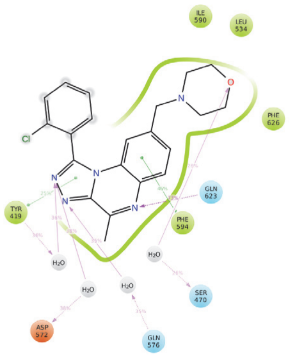	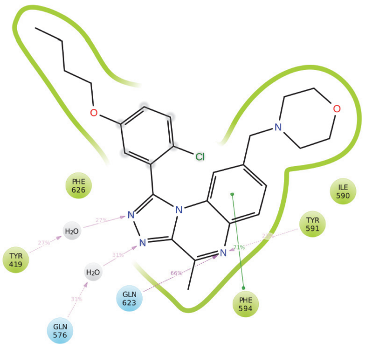
